# Molecular Identification of Birds: Performance of Distance-Based DNA Barcoding in Three Genes to Delimit Parapatric Species

**DOI:** 10.1371/journal.pone.0004119

**Published:** 2009-01-07

**Authors:** Mansour Aliabadian, Mohammad Kaboli, Vincent Nijman, Miguel Vences

**Affiliations:** 1 Institute for Biodiversity and Ecosystem Dynamics and Zoological Museum, University of Amsterdam, Amsterdam, The Netherlands; 2 Department of Biology, Faculty of Sciences, Ferdowsi University of Mashhad, Mashhad, Iran; 3 Department of Fishery and Environment, Faculty of Natural Resources, University of Tehran, Tehran, Iran; 4 School of Social Sciences and Law, Oxford Brookes University, Oxford, United Kingdom; 5 Division of Evolutionary Biology, Zoological Institute, Technical University of Braunschweig, Braunschweig, Germany; University of Otago, New Zealand

## Abstract

**Background:**

DNA barcoding based on the mitochondrial cytochrome oxidase subunit I gene (*cox1* or COI) has been successful in species identification across a wide array of taxa but in some cases failed to delimit the species boundaries of closely allied allopatric species or of hybridising sister species.

**Methodology/Principal Findings:**

In this study we extend the sample size of prior studies in birds for *cox1* (2776 sequences, 756 species) and target especially species that are known to occur parapatrically, and/or are known to hybridise, on a Holarctic scale. In order to obtain a larger set of taxa (altogether 2719 species), we include also DNA sequences of two other mitochondrial genes: cytochrome *b* (*cob*) (4614 sequences, 2087 species) and *16S* (708 sequences, 498 species). Our results confirm the existence of a wide gap between intra- and interspecies divergences for both *cox1* and *cob*, and indicate that distance-based DNA barcoding provides sufficient information to identify and delineate bird species in 98% of all possible pairwise comparisons. This DNA barcoding gap was not statistically influenced by the number of individuals sequenced per species. However, most of the hybridising parapatric species pairs have average divergences intermediate between intraspecific and interspecific distances for both *cox1* and *cob*.

**Conclusions/Significance:**

DNA barcoding, if used as a tool for species discovery, would thus fail to identify hybridising parapatric species pairs. However, most of them can probably still assigned to known species by character-based approaches, although development of complementary nuclear markers will be necessary to account for mitochondrial introgression in hybridising species.

## Introduction

Mitochondrial DNA (mtDNA) markers have been widely applied in molecular phylogenetic studies, but deciding which mtDNA genes to use for the identification of species remains an important issue [1]–[3] because different parts of the mtDNA genome evolve at different mutation rates [4], [5]. The choice of a suitable gene with high phylogenetic resolution will be more crucial when evaluating species delimitation of recently diverged species. MtDNA, with rapid pace of sequence changes, regularly shows pronounced divergences between closely related species [1], [6] but concern has been expressed that mtDNA sequence differences among such closely related species will often be too small to allow their discrimination, and the problem will be further accentuated by phenomena of ancient sharing of haplotype polymorphisms and by introgression (e.g. [7]).

Recent studies suggest that sequences of the mitochondrial cytochrome oxidase subunit I (*cox1 or COI*) could serve as a fast and accurate marker for the identification of animal species, and for the discovery of new species across the tree of life [8], a procedure for which the term DNA barcoding has been coined. A first major study investigated sequence variation of 25% of the species of North American breeding birds (260 species) [9]. Variation of *cox1* sequences within species was an average of 20 times smaller than between species, and there was a clear gap between intra- and interspecific variation. Utilizing this barcoding gap, a standard sequence threshold was proposed to define species boundaries of around 10 times the mean intraspecific variation for the group under study [9]. DNA barcoding based on *cox1* has been successful in species identification across a wide array of taxa [9]–[12]. For invertebrates, it has however been argued [13] that the barcoding gap may be an artifact of insufficient sampling across taxa.

In general, it is especially with pairs or complexes of closely related and potentially hybridising species where DNA barcoding can be expected to encounter problems [14]. In particular the existence of so-called parapatric species may pose a challenge for DNA barcoding. For birds, parapatric species are defined as species with contiguous or narrow overlap zones, excluding each other geographically; these species may or may not hybridise, and may or may not represent sister species, but phylogenetic data are incomplete thus far [15]. Only few DNA barcoding studies have focused on potentially hybridising bird species thus far [12], [16], which probably in part is due to the lack of *cox1* sequences for crucial taxa. Especially the parapatric species pairs from the Palearctic have remained largely unstudied in this respect.

Data are available for 39 pairs of proven sister species of North American birds, most of which hybridise; these have K2P (Kimura two-parameter) mtDNA distances of 0.07% to 8.2%, with an average of 1.9% [12]. For 29 out of these 39 species pairs the sequence divergences was equal or lower than the suggested *cox1* threshold (2.7%) [9]. Building upon this previous work, [9], Kerr *et al.* (2007) [16], working with a larger number of species and samples, found significantly smaller amounts of interspecific variation between closely related allopatric bird taxa (that often are known to hybridise), potentially compromising the universal applicability of *cox1* DNA barcoding.

Besides the *cox1* gene, other mitochondrial markers also have been widely sequenced across vertebrates for their utility in phylogenetics or to complement *cox1* in DNA barcoding. In amphibians the *16S* ribosomal RNA gene (*16S*) has been suggested as a complementary DNA barcoding marker [17]. Another protein coding gene, cytochrome b (*cob*), has also been suggested as a marker to determine species boundaries [18]–[20]. Birds are among the most intensively sequenced taxa for *cox1* and *cob*, and there is a reasonable dataset available for *16S*. Taken together, sequences of these three genes are available for a significant proportion of worldwide species diversity of birds. Furthermore, birds are taxonomically one of the best studied animal groups which indicates that a relatively low proportion of unknown, cryptic species is to be expected, and that taxonomic misidentifications are relatively rare, giving a reasonable degree of confidence in the specific identity of published DNA sequences.

We here make use of the availability of *cob* and *16S* data to combine these with *cox1* sequences into the largest taxon set that so far has been assessed for mitochondrial divergences at different taxonomic levels. We specifically aimed to provide novel data on (a) a possible dependence of the barcoding gap between intra- and interspecific divergences from the number of sequences per species, (b) a comparison of levels of pairwise divergences among species in the same genus vs. species in different genera, and, especially, (c) the utility of DNA barcoding to discern among mainly Palearctic hybridising parapatric species.

## Results

For none of the three genes was mean divergence within species significantly related to the sample sizes per species, as revealed by regression analysis (*cox1*: R^2^ = 0.001, *p* = 0.465, *16S*: R^2^ = 0.001, *p* = 0.465, *cob*: R^2^ = 0.001, *p* = 0.338) (Figure S1). In general, intraspecific K2P distances for the three genes ranged from zero to 17.9% (*cox1*: 0–7.3%, *16S*: 0–6.2%, and *cob*: 0–17.9) and intrageneric K2P distances ranged from zero to 20.1% (*cox1*: 0–18.9%, *16S*: 0–13.3%, and *cob*: 0–20.1). The lower range of values may be an effect of misnamed or misidentified taxa in GenBank, or may be real (as in the case of several taxa that form a so-called ring-species: [21]–[23]. Similarly, we strongly suspect that many of the the highest intraspecific distances are due to wrongly determined samples, or pseudogene sequences, recovered from Genbank. In total, only 134 out of 31,773 *cob* intraspecific K2P values were above 7.4% (and thus 10-fold higher than average intraspecific divergence, see below), indicating that these possibly wrong comparisons (affecting 63 species) will have a very limited effect on subsequent calculations.

### Cox1 gene

**Figure 1:**
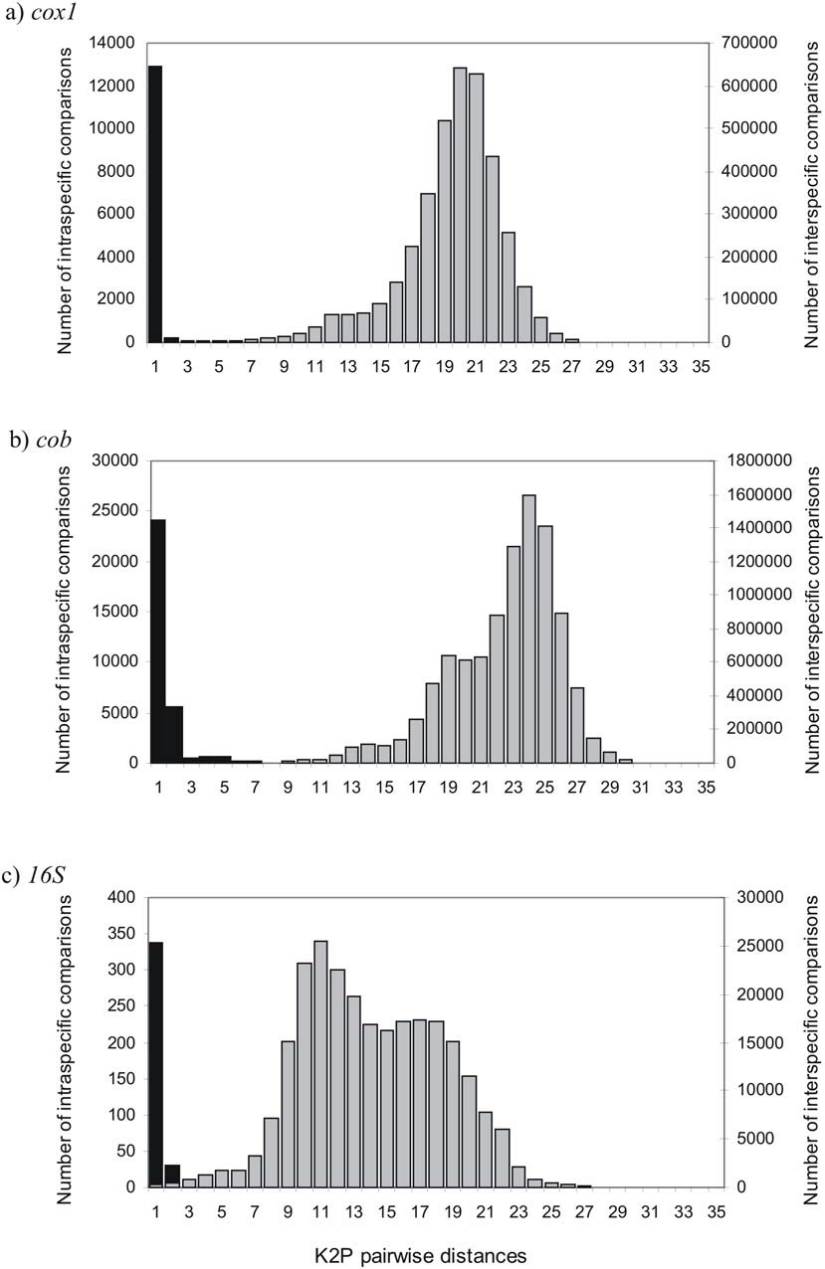
K2P pairwise distances in (a) *cox1*, (b) *cob*, and (c) *16S* genes. Black bars are comparisons among intraspecific sequences (left axis) and grey bars represent comparisons among different species (right axis).

Intraspecific K2P distances for species with ≥2 sequences (mean = 4.51, range = 2–122, n = 566 species) averaged 0.24% (SD = 0.59%). Intrageneric K2P distances are some 24-fold higher (5.97±3.55) than the mean intraspecific K2P distances (Figure 1a, Table S1). Mean divergences within families and orders were 11.46% (SD = 3.06%) and 15.80% (SD = 3.35%) respectively (Figure S2a).

**Figure 2:**
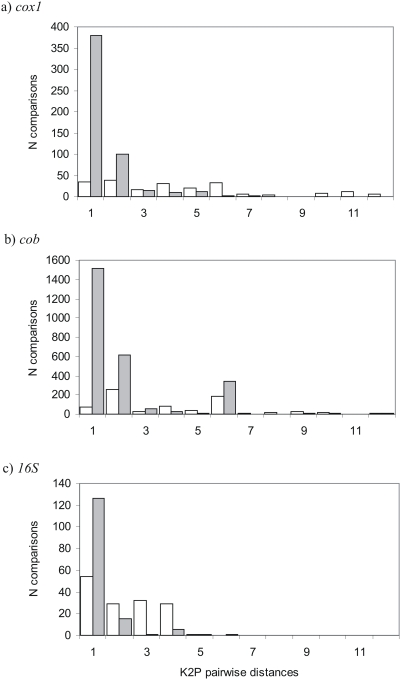
K2P Pairwise comparisons among parapatric bird species in (a) *cox1*, (b) *cob*, and (c) *16S* genes. White bars are comparisons between pairs of parapatric species and grey bars are comparisons within each of these species.

K2P distances within 64 parapatric species (comparing species occurring parapatrically with at least one other related bird species) with >2 sequences (mean = 3.1, range = 2–8) averaged 0.49±0.87% (Figure 2a). K2P distances between species in parapatric species pairs averaged 3.64±3.29% with significant differences between those species that do hybridise (3.35±3.35%) and those that do not hybridise (5.99±4.24%) (*p*>0.001 Table 1, 2). K2P distances between species in hybridising species pairs were significantly larger than intraspecific distances and smaller than intrageneric K2P distances for all species (*p*<0.001, Table 2).

**Table 1:** Mean K2P pairwise distances between species in parapatric species pairs in three genes, *cox1*, *cob*, and *16S*.

species pairs	K2P distances		
	*cox1*	*cob*	*16s*
**Parapatric species pairs that do hybridise**			
*Acrocephalus stentoreus/Acrocephalus arundinaceus*		5.5	
*Alectoris chukar/Alectoris rufa*		5	
*Alectoris graeca/Alectoris chukar*		5	
*Alectoris magna/Alectoris chukar*		5	
*Anthus pratensis/Anthus spinoletta*	3.6		
*Anthus spinoletta/Anthus pratensis*		5	1.7
*Aquila pomarina/Aquila clanga*		2.7	
*Archilochus alexandri/Archilochus colubris*	1.6		
*Callipepla californica/Callipepla gambelii*	2.1		
*Campylorhynchus zonatus/Campylorhynchus albobrunnea*		4	
*Carpodacus cassinii/Carpodacus purpureus*	5.8		
*Chrysolophus pictus/Chrysolophus amherstiae*		2.3	
*Contopus sordidulus/Contopus virens*	11.1		
*Contopus virens/Contopus sordidulus*		2	
*Coturnix japonica/Coturnix coturnix*			3
*Crossoptilon auritum/Crossoptilon crossoptilon*		3	
*Delichon urbicum/Delichon dasypus*		8.5	
*Dendrocopos major/Dendrocopos syriacus*	4.1		
*Dendroica occidentalis/Dendroica townsendi*	0.4		
*Emberiza melanocephala/Emberiza bruniceps*	6.5		1.2
*Eremophila bilopha/Eremophila alpestris*	4.2		0.6
*Galerida theklae/Galerida cristata*		7.9	0.6
*Gallus gallus/Gallus sonneratii*	2.1	3	
*Gavia immer/Gavia adamsii*	0.6		
*Hippolais polyglotta/Hippolais icterina*		7	2.6
*Lanius collurio / Lanius isabellinus*	2.4	1	1.2
*Lanius collurio/Lanius cristatus*		9	1.2
*Lanius isabellinus/Lanius cristatus*		8.2	
*Lanius isabellinus/Lanius vittatus*		8	
*Lanius schach/Lanius tephronothus*			1.7
*Lanius vittatus/Lanius collurioides*		6	
*Larus glaucoides/Larus thayeri*	0.1		
*Larus argentatus/Larus cachinnans*	0.3	0.8	0.1
*Leucopternis melanops/Leucopternis kuhli*		2	
*Lophura nycthemera/Lophura leucomelanos*		3	
*Luscinia megarhynchos/Luscinia luscinia*	5.1		0.8
*Melanerpes aurifrons/Melanerpes carolinus*	4.5		
*Melanerpes aurifrons/Melanerpes uropygialis*	6		
*Oenanthe hispanica/Oenanthe pleschanka*	0.1		1
*Passer indicus/Passer domesticus*			0.5
*Passerina amoena/Passerina cyanea*	9.4	7.7	
*Pheucticus ludovicianus/Pheucticus melanocephalus*	5.1		
*Phylloscopus sindianus/Phylloscopus collybita*	7.2	4	2.2
*Phylloscopus sibilatrix/Phylloscopus bonelli*	7.4	9.3	2.3
*Picoides nuttallii/Picoides scalaris*	1.4	9	
*Piranga ludoviciana/Piranga bidentata*		4.8	
*Plegadis falcinellus/Plegadis chihi*	0.9		
*Pluvialis dominica/Pluvialis fulva*	4.8		
*Poecile carolinensis/Poecile atricapillus*		6	
*Poephila cincta/Poephila acuticauda*		3	
*Polioptila californica/Polioptila melanura*	2		
*Selasphorus rufus/Selasphorus sasin*	2.4		
*Sialia sialis/Sialia currucoides*		6.7	
*Sphyrapicus ruber/Sphyrapicus varius*	2.8		
*Streptopelia vinacea/Streptopelia capicola*		2	
*Sturnus unicolor/Sturnus vulgaris*			0.5
*Tragopan blythii/Tragopan temminckii*		7	
*Turdus ruficollis/Turdus naumanni*			0
*Vireo cassinii/Vireo solitarius*	2.1	3	
**Parapatric species pairs that do not hybridise**			
*Alectoris philbyi/Alectoris melanocephala*		7	
*Anthus spinoletta/Anthus rubescens*	5		
*Campyloramphus trochilirostris/Campyloramphus procurvoides*	5		
*Circaetus cinerascens/Circaetus fasciolatus*		4	
*Corythaixoides concolor/Corythaixoides personata*		6	
*Crax globulosa/Crax alector*		4	
*Crax rubra/Crax alberti*		5	
*Crinifer piscator/Crinifer zonurus*		5	
*Emberiza caesia/Emberiza hortolana*			0.1
*Emberiza hortulana/Emberiza buchanani*	5.8		1.2
*Gavia arctica/Gavia pacifica*	4.7		
*Geospiza difficilis/Geospiza fuliginosa*			1
*Hippolais olivetorum/Hippolais languida*		9	
*Hirundo aethiopica/Hirundo lucida*		2	
*Hirundo albigularis/Hirundo angolensis*		9	
*Hirundo nigrita/Hirundo smithii*		9	
*Locustella lanceolata/Locustella fluviatilis*		11	2.7
*Melanocorypha calandra/Melanocorypha bimaculata*	9.9		1.3
*Melithreptus albogularis/Melithreptus lunatus*		10	
*Motacilla alba/Motacilla madaraspatensis*		5.9	
*Musophaga violacea/Musophaga rossae*		6	
*Parus xanthogenys/Parus spilonotus*		5	
*Ramphastos brevis/Ramphastos vitellinus*		3.9	
*Selenidera reinwardtii/Selenidera gouldii*		5	
*Sitta neumayer/Sitta tephronota*	0.9		1.1
*Streptopelia orientalis/Streptopelia turtur*		5	
*Sylvia mystacea/Sylvia melanocephala*	9.8	5	2.3
*Syrmaticus reevesi/Syrmaticus ellioti*		8.5	
*Tragopan satyra/Tragopan blythii*		6	
*Tragopan temminckii/Tragopan satyra*		7	
*Trogon melanurus/Trogon comptus*		8	
*Turdus pallidus/Turdus obscurus*		2	
*Veniliornis cassini/Veniliornis affinis*	5.7		
*Zosterops senegalensis/Zosterops pallidus*		5	

**Table 2:** K2P distances of species in parapatric species pairs and among non-parapatric species, comparing parapatric species pairs that do hybridise with those that do not, and with intrageneric (excluding parapatric species), intraspecific (excluding parapatric species), intraspecific in hybridising parapatric species, and intraspecific in non-hybridising parapatric species (** = 0.01, t-test).

Comparisons	*cox1*	*cob*	*16S*
	K2P Mean±std (N)	K2P Mean±std (N)	K2P Mean±std (N)
Between hybridising parapatric species	3.35±3.05 (191)	2.76±2.28 (662)	1.36±1.23 (119)
Between non-hybridising parapatric species	5.99±4.24 (23)**	6.17±2.36 (69)**	1.01±0.79 (25)
Within all genera (excl. parapatric species)	5.99±3.54 (27162)**	8.2±3.75 (40555)**	3.26±1.91 (1731)**
Within all species (excl. parapatric species)	0.23±0.57 (12924)**	0.72±1.15 (29191)**	0.57±1.18 (246)**
Within hybridising parapatric species	0.49±0.87 (496)**	1.01±1.67 (2488)**	0.28±0.68 (128)**
Within non-hybridising parapatric species	0.37±0.29 (24)**	2.22±2.56 (105)	0.63±1.37 (22)**

Presented are mean±standard deviation (number of comparisons).

### Cob gene

Intraspecific K2P distances for species with ≥2 sequences (mean = 4.64, range = 2–127, n = 656 species) averaged 0.74% (SD = 1.21%). Intrageneric K2P distances were some 11-fold higher (8.11±3.80) than the mean intraspecific K2P distances (Figure 1b, Table S1). Mean divergences within families and orders were 13.97% (S = 3.13%) and 19.50% (SD = 3.64%) respectively (Figure S2b).

K2P distances within 60 parapatric species with ≥2 sequences (mean = 3.51, range = 2–20) averaged 1.07±1.74% (Figure 2b). K2P distances between species in parapatric species pairs averaged 3.08±2.50, with a significant difference between those species that do hybridise and those that do not hybridise (*p*<0.001, Table 1, 2). Intrageneric K2P distances between species in hybrid species pairs were significantly different from intraspecific and intrageneric K2P distances for all species (*p*<0.001, Table 2).

### 16S gene

Intraspecific K2P distances for species with ≥2 sequences (mean = 2.67, range = 2–12, n = 125 species) averaged 0.48% (SD = 1.06%). Intrageneric K2P distances were some seven fold higher (3.13±1.92) than the mean intraspecific K2P distances (Figure 1c, Table S1). Mean divergences within families and orders were 6.51% (SD = 2.45%) and 10.69% (SD = 2.37%) respectively (Figure S2c).

K2P distancess within 46 parapatric species with ≥2 sequences (mean = 2.22, range = 2–12) averaged 0.33±0.82% (Figure 2c). K2P distances between species in parapatric species pairs averaged 1.30±1.17, with no significant difference between those species that do hybridise and those that do not hybridise (*p* = 0.078 Table 1, 2). K2P distances between species in hybridising species pairs were significantly different from intraspecific and intrageneric K2P distances for all species (*p*<0.001, Table 2).

## Discussion

### DNA barcoding efficiency of cox1 vs. cob and 16S

The accuracy of distance-based DNA barcoding depends particularly on the extent of the separation between intra- and interspecific divergence in the selected marker. The ideal world for barcoding lacks any overlap between these two values. By including *cob* and *16S* in our analysis, besides *cox1*, we have been able to test the overlap between inter- and intraspecific mitochondrial distances in a much wider array of taxa than previous analysis [9], [16]. Furthermore, by specifically targeting parapatric and hybridising bird taxa which potentially are particularly problematic in DNA barcoding [14], we here provide a more stable basis to test the performance of mitochondrial DNA barcoding in these species.

As apparent from Figure 1 (and Figure S2abc, Supporting information for comparisons within higher taxonomy levels for each gene) a wide gap exist between intra- and interspecies divergences for both *cox1* and *cob* genes if all taxa within genera are compared, whereas this gap is less apparent for *16S*. This indicates that mitochondrial rRNA genes may be less suitable for bird species identification despite their many other advantages like universal primer applicability [17]. In the following we thus do not use the *16S* data further to discuss the distribution of sequence divergences among birds, but report the *cob* data as a complement to *cox1* in order to base our comparisons on the maximum available number of taxa.

### Effects of sample size on DNA barcoding gap

The intrageneric distances of *cox1*, on average, were 24-fold higher that the sequence divergences within species (0.24 vs 5.97%). In *cob* the intrageneric distances were on average 11-fold higher than the sequence divergences within species (0.74 vs 8.11%). The slight differences between the two data sets may be due to a different composition of the data (intraspecific comparisons in *cob* possibly based on samples from more distant localities).

These values are not too different from those obtained in an initial effort to test *cox1* DNA barcoding in birds [9], based on two or more individuals for 130 species (0.27% vs. 7.93%). It has been argued that this alleged gap will considerably lower down if more individuals per species are sampled and when a large proportion of closely related taxa are included [14], [24]. This effect was however not observed in a subsequent study [16] that analysed an average 4.1 individuals per species. Our study confirms this trend. The barcoding gap was apparent in the *cox1* dataset with, on average, 4.51 sequences per species, and also in the independent *cob* dataset with 4.64 sequences per species. Furthermore, our regression analyses found no dependence of intraspecific divergences from the number of individuals per species included in the analysis, neither in *cox1* nor in *cob*.

The present study compared the divergence of intraspecific sequences from specimens that in a high proportion originated from widely separated geographic regions, and confirmed that *cox1* sequence variation was able to identify more than 98% of the pairwise sequence divergences correctly as corresponding to variation within species. In contrast, 20% of the pairwise comparisons within genera (intrageneric sequences distances) were lower than 0.24%. Intraspecific variation identified in this study was similar to that in North American breeding birds: 0.24% vs. 0.23% and 0.27% [9], [16]. These values are lower than in most other animal groups: e.g., 0.60% in Guyanese bats [25], 0.46% in Lepidoptera [26], 0.39% in marine fishes [27], 3–4% in *Aneides* salamanders and mantellid frogs [17].

### DNA barcoding in parapatric bird species

While the barcoding gap appears to hold for overall comparisons among birds even if larger numbers of individuals are included, a more critical issue is that of distinguishing related combinations of species [14]. In such species complexes, the barcoding gap may not exist, and this effect may be diluted in overall comparisons of large numbers of taxa. Numerous DNA barcoding studies conducted thus far revealed that more than 90% of species under study could be identified by this method. For example, 93% of studied species of Guyanan bats and 95% of North American bird species could be allocated correctly [25], [16]. The cases where barcodes failed to separate species involved either closely allied allopatric taxa whose status, as distinct species, is uncertain, or sister taxa that hybridise. However, coalescent and character-based approaches are effective in closely related species, non-hybridising species of birds [10].

Our study showed that a high proportion of hybridising parapatric species cannot be distinguished by the suggested distance-based threshold value in DNA barcoding. The proportion was 48% (14/29) in the *cox1* dataset and 78% (25/32) in the *cob* dataset. These different values probably were not due to different properties of the analysed genes but to different taxa included, and possibly to a higher degree of misidentified taxa (as taken from Genbank) in the *cob* dataset. Of the parapatric species pairs that do not hybridise, 14% (1/7) and 73% (19/26) did not meet the threshold for *cox1* and *cob* genes respectively.

Based on published [e.g., 28] and unpublished estimates (own data) the percentage of parapatric species that hybridise in the Palearctic Region is 60%, which corresponds to 10–18% of all species: [29], [30] Using these values, plus the global number of parapatric species [15], [28] and the proportion of parapatric species that show only a small amount of genetic divergence, we can estimate that between 250 (based on *cox1*) and 650 (*cob*) parapatric species of birds are not distinguishable by the barcoding gap. This represents some 2.5–6.0% of the total number of species. If DNA barcoding would be used as a tool for species discovery, it would fail to identify these species.

However, especially in a well-known group such as birds, DNA barcoding is usually used for assigning individuals to known species. In these cases, most of the parapatric species could probably be still correctly identified, depending on the origin of the low divergences: (a) mitochondrial introgression due to hybridisation, or incomplete lineage sorting, which would cause some individuals in one species being closer to individuals of another species than to conspecifics; or (b) an origin of parapatric species pairs by recent speciation, and therefore overall low genetic divergences between them. Our data set does not allow distinguishing between these two causes, but further research into this question would be useful to understand the processes influencing the perspectives and reliability of DNA barcoding in birds. Furthermore, we should keep in mind that the named taxa also might be incorrectly classified, i.e., should be lumped into single species. If most of the problematic cases refer to introgression and incomplete lineage sorting, then nuclear markers need to be developed to reliably discern between the affected species [31]. If recent speciation and generally low genetic distances (but reciprocally monophyletic haplotype lineages) are involved, then character based DNA barcoding may be more appropriate and would allow to sidestep the problem to find appropriate threshold values by searching “barcoding gaps”. In any case, where not only species identification but species discovery is concerned, it is clear that DNA barcoding should be used as only one (in many groups the first preliminary) step in the recognition, diagnosis and description of species in terms of integrative taxonomy (e.g. [32]).

## Materials and Methods

### Data sampling

The study was carried out in compliance with the institutional guidelines on animal husbandry and experiments of the the Zoological Museum of the University of Amsterdam. In addition, the authorization for the experiments was given by the Iranian (*permission number*: 3–5360) and Moroccan (*p.n.*: 04666 DCRF/CPB/PFF) authorities.

We sequenced 210 individuals of 145 nominal species for DNA sequences of *cox1* and *16S* gene fragments, of which 31 and 46 species were parapatric species for *cox1* and *16S* respectively. Parapatric species are defined as species that have at least one other closely-related species which inhabits a continuous range, the two species excluding each other geographically [15], [33]. The range boundary between the two taxa has no dispersal barrier, allowing parapatric species to hybridise and display intergradation in their contact zones, yet they maintain distinct outside of these zones [34], [35].

DNA was extracted from tissue or blood samples using DNeasy Tissue Kits (QIAGEN) following the manufacturer's protocol. Polymerase chain reactions (PCR) and sequencing reactions follow protocols described by Aliabadian *et al.* (2007) [36] which can be summarized as follows. A fragment of *cox1*, was sequenced using two primer combinations that amplify a region of 612 bp starting from the 5′ terminus of the mitochondrial *cox1* gene: BirdF1 (5′ -TTC TCC AAC CAC AAA GAC ATT GGC AC -3′), BirdR1 (5′ -ACG TGG GAG ATA ATT CCA EET CCT G- 3′), and BirdR2 (5′ -ACT ACA TGT GAG ATG ATT CCG AAT CCA G - 3′) [9]. A fragment of *16S* was sequenced for the same individuals using 16SA-L (light chain; 5′-CGC CTG TTT ATC AAA AAC AT-3′) and 16SB-H (heavy chain; 5′-CCG GTC TGA ACT CAG ATC ACG T-3′) [37]. PCR products were cleaned using QIAquick PCR Purification Kit (Qiagen). Sequencing reactions were resolved on ABI 3100 or ABI 3730 automated DNA sequencers. Genbank accession numbers of newly determined sequences are FJ465179–FJ465383 and are listed in detail in Table S2).

Our data set was complemented by *cox1* and *16S* sequences from GenBank, as available on 1 July 2006). For *cox1*, additional sequences were included from the Barcode of Life Data Systems website (http://www.barcodinglife.org/, as accessed on 1 July 2006). Sequences were included provided they had a length of >612 (*cox1*) and >538 bp (*16S*) homologous to our sequences, with no more than 50 ambiguous or missing nucleotides. *Cob* sequences with a length of >1000 bp and no more than 50 ambiguous or missing nucleotides were retrieved from GenBank as well. Because of a probably high prevalence of misidentification, erroneous sequences or NUMTs in the Genbank sequences, we submitted these to a rigurous quality control. All sequences per gene were aligned and a Neighbor-joining tree produced. We identified, in this tree, all sequences clustering far from their known taxonomic or phylogenetic position, or characterized by extreme branch lengths, and omitted these sequences from further analysis. For *cob*, the gene were the largest data set (over 10,000 sequences) was initially downloaded, less than half of these were of sufficient length and quality.

All sequences were aligned using Muscle, a multiple alignment software for protein and nucleotide sequences which allows multiple sequence comparison by log-expectation [38]. Probably erroneous sequences (with highly unlikely positions or extreme branch lengths, based on a neighbour-joining tree calculated with all sequences) were identified by eye and omitted. A total of 2776 sequences (756 nominal species) were kept for *cox1*, 708 (498 species) for *16S*, and 4614 (2087 species) for *cob* (Table 3), and altogether 2719 species were included for at least one of the genes (Table S2). Among conspecific sequences, we verified that for many species, samples from distant localities were included and our analysis is thus not based on including only samples from the same locality and population.

**Table 3:** Number of individuals and taxa employed in this study.

	Individuals	species	genera	families	orders
all birds		9721	2161	244	25
*cox1*	2776	756	329	75	20
*cob*	4614	2087	890	114	24
*16S*	708	498	270	91	25

### Data analysis

Genetic distances were calculated to quantify sequence divergences among individuals using Kimura's (1980) [39] two-parameter (K2P) models, theta, as implemented in MEGA 3.1 [40]. The K2P distance is the most effective model when genetic distances are low [8]. K2P distances were calculated at all taxonomic levels, intraspecific, intrageneric, intrafamilial, intraordinal, and, separately, between species in parapatric species pairs, following a published taxonomy [41] and unpublished data by CS Roselaar. For calculation involving higher taxonomic levels, pairwise comparisons of the previous levels were excluded (e.g., in comparisons of intrageneric, pairwise distances of intraspecific were removed, and only pairwise distances of those samples to other species were used).

Average K2P distances were computed based on pairwise comparisons of all sequences for each species, and each pair of parapatric species. To calculate intra- and interspecific pairwise distances, based on output matrix of MEGA 3.1, we wrote a converter program SPD 1.1) in C language (this program will be available from the authors upon request).

Altogether 3,823,995, 9,952,500, and 250,257 pairwise distances were compared in this study for, *cox1*, *cob*, *and 16S* respectively. NJ trees of K2P distances showing inter- and intra specific variations were constructed using MEGA 3.1 (not shown here). A regression analysis was employed to assess the effect of sample size on intraspecific divergences for each gene using SPSS for Windows, version 11.

## Supporting Information

Figure S1 The relationship between mean intraspecific variations (K2P) and the number of individuals analysed for each species. Black squares: cox1 (adjusted R^2^ = 0.001, P = 0.465). Grey dots: cob (adjusted R^2^ = 0.001, P = 0.338)
(0.41 MB TIF)

Figure S2 Comparisons of K2P pairwise distances in (A) cox1, (B) cob, and (C) 16S genes in birds. Mean (±SD). K2P distances are compared within various level of taxonomic hierarchy for three genes.
(3.89 MB TIF)

Table S1 K2P Mean intraspecific distances for cox1, cob, and 16S.
(1.23 MB DOC)

Table S2 List of all samples that have been sequenced in this study, with voucher numbers and collection localities
(0.24 MB DOC)
