# Supplementary figures and images for: Molecular Identification of Birds: Performance of Distance-Based DNA Barcoding in Three Genes to Delimit Parapatric Species
Source: PLoS One. 2009 Jan 7;4(1):e4119. doi: 10.1371/journal.pone.0004119 (PMC2612741; doi:10.1371/journal.pone.0004119)

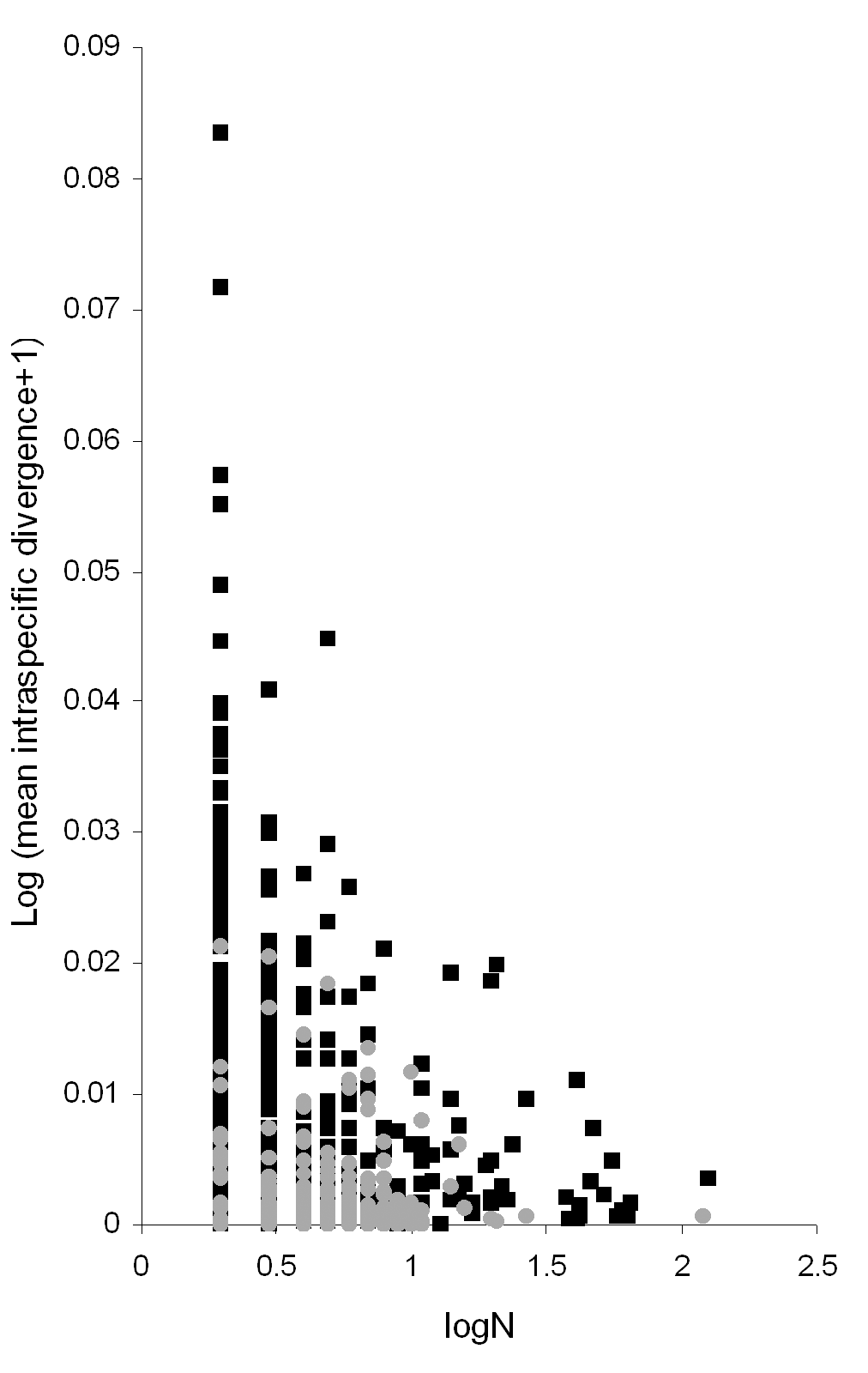

Supplement: Figure S1 — The relationship between mean intraspecific variations (K2P) and the number of individuals analysed for each species. Black squares: cox1 (adjusted R2 = 0.001, P = 0.465). Grey dots: cob (adjusted R2 = 0.001, P = 0.338) (0.41 MB TIF) [file pone.0004119.s001.tif]

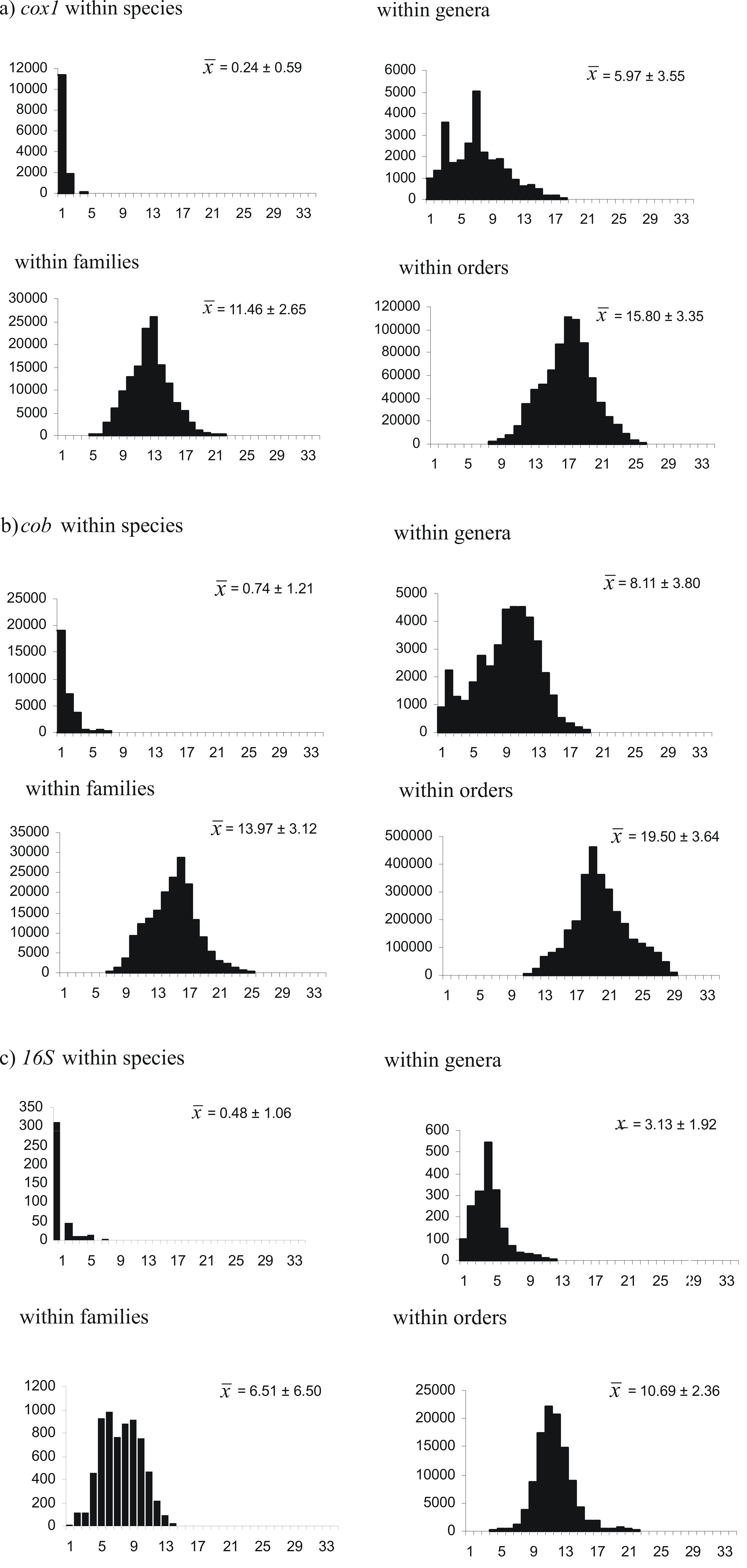

Supplement: Figure S2 — Comparisons of K2P pairwise distances in (A) cox1, (B) cob, and (C) 16S genes in birds. Mean (±SD). K2P distances are compared within various level of taxonomic hierarchy for three genes. (3.89 MB TIF) [file pone.0004119.s002.tif]
