# Supplementary material for: Molecular Identification of Birds: Performance of Distance-Based DNA Barcoding in Three Genes to Delimit Parapatric Species
Source: PLoS One. 2009 Jan 7;4(1):e4119. doi: 10.1371/journal.pone.0004119 (PMC2612741; doi:10.1371/journal.pone.0004119)
Supplement: Table S1 — K2P Mean intraspecific distances for cox1, cob, and 16S. (1.23 MB DOC) [file pone.0004119.s003.doc]

**Table S1.**

| Scientific name | Number of individual | %Mean divergence *cox1* | %Mean divergence *16s* | %Mean divergence *cob* |
| --- | --- | --- | --- | --- |
| *Acanthiza apicalis* | 0/0/2 | - | - | 0.0 |
| *Acanthiza chrysorrhoa* | 0/2/2 | - | 0.2 | 0.1 |
| *Acanthiza ewingii* | 0/3/0 | - | 0.0 | - |
| *Acanthiza inornata* | 0/2/0 | - | 0.0 | - |
| *Acanthiza iredalei* | 0/3/0 | - | 0.0 | - |
| *Acanthiza katherina* | 0/2/0 | - | 0.4 | - |
| *Acanthiza lineata* | 0/2/0 | - | 0.0 | - |
| *Acanthiza nana* | 0/2/0 | - | 0.0 | - |
| *Acanthiza pusilla* | 0/2/0 | - | 0.2 | - |
| *Acanthiza reguloides* | 0/4/0 | - | 0.0 | - |
| *Acanthiza robustirostris* | 0/2/0 | - | 0.0 | - |
| *Acanthiza uropygialis* | 0/2/0 | - | 0.0 | - |
| *Accipiter cooperii* | 5/0/0 | 0.0 | - | - |
| *Accipiter gentilis* | 6/0/2 | 0.1 | - | 0.2 |
| *Accipiter striatus* | 5/0/0 | 0.0 | - | - |
| *Acridotheres tristis* | 4/2/0 | 0.0 | 0.2 | - |
| *Acrocephalus agricola* | 0/0/6 | - | - | 2.9 |
| *Acrocephalus arundinaceus* | 0/0/3 | - | - | 0.3 |
| *Acrocephalus australis* | 0/0/3 | - | - | 0.5 |
| *Acrocephalus avicenniae* | 0/0/2 | - | - | 0.0 |
| *Acrocephalus baeticatus* | 0/0/4 | - | - | 2.9 |
| *Acrocephalus bistrigiceps* | 0/0/6 | - | - | 2.1 |
| *Acrocephalus concinens* | 0/0/3 | - | - | 0.3 |
| *Acrocephalus dumetorum* | 0/0/3 | - | - | 1.5 |
| *Acrocephalus gracilirostris* | 0/0/8 | - | - | 1.7 |
| *Acrocephalus griseldis* | 0/0/3 | - | - | 0.2 |
| *Acrocephalus melanopogon* | 0/0/11 | - | - | 1.4 |
| *Acrocephalus newtoni* | 0/0/2 | - | - | 1.4 |
| *Acrocephalus orientalis* | 0/0/14 | - | - | 0.4 |
| *Acrocephalus paludicola* | 0/0/3 | - | - | 0.7 |
| *Acrocephalus palustris* | 0/0/2 | - | - | 0.1 |
| *Acrocephalus rufescens* | 0/0/2 | - | - | 0.4 |
| *Acrocephalus schoenobaenus* | 0/0/7 | - | - | 0.5 |
| *Acrocephalus scirpaceus* | 0/0/11 | - | - | 2.4 |
| *Acrocephalus sechellensis* | 0/0/2 | - | - | 0.2 |
| *Acrocephalus stentoreus* | 0/0/4 | - | - | 1.4 |
| *Acrocephalus tangorum* | 0/0/3 | - | - | 0.7 |
| *Acrocephalus vaughani* | 0/0/3 | - | - | 0.4 |
| *Actitis macularia* | 6/0/0 | 0.0 | - | - |
| *Aechmophorus clarkii* | 2/0/0 | 0.0 | - | - |
| *Aegithalos caudatus* | 0/0/4 | - | - | 0.1 |
| *Aegolius acadicus* | 6/0/0 | 0.0 | - | - |
| *Aegolius funereus* | 2/0/0 | 0.3 | - | - |
| *Aegolius funereus* | 0/0/5 | - | - | 0.5 |
| *Aegypius monachus* | 0/0/2 | - | - | 1.6 |
| *Aerodramus elaphrus* | 0/0/3 | - | - | 0.1 |
| *Aerodramus francicus* | 0/0/2 | - | - | 0.0 |
| *Aerodramus fuciphagus* | 0/0/9 | - | - | 1.7 |
| *Aerodramus maximus* | 0/0/11 | - | - | 1.2 |
| *Aerodramus salangana* | 0/0/3 | - | - | 0.0 |
| *Aerodramus spodiopygius* | 0/0/4 | - | - | 1.7 |
| *Aerodramus terraereginae* | 0/0/3 | - | - | 0.1 |
| *Aerodramus vanikorensis* | 0/0/3 | - | - | 1.7 |
| *Aeronautes saxatalis* | 3/0/0 | 0.0 | - | - |
| *Aethia pusilla* | 2/0/0 | 0.0 | - | - |
| *Afrotis afraoides* | 0/0/5 | - | - | 0.1 |
| *Agelaius phoeniceus* | 6/0/0 | 0.2 | - | - |
| *Aimophila cassinii* | 5/0/0 | 0.0 | - | - |
| *Aimophila ruficeps* | 2/0/0 | 0.1 | - | - |
| *Aix sponsa* | 9/0/0 | 0.0 | - | - |
| *Alaemon alaudipes* | 0/2/3 | - | 0.0 | 0.3 |
| *Alauda arvensis* | 4/3/0 | 0.4 | 0.0 | - |
| *Alca torda* | 6/0/0 | 0.1 | - | - |
| *Alectoris chukar* | 3/0/9 | 0.2 | - | 0.4 |
| *Alectoris rufa* | 0/0/15 | - | - | 1.8 |
| *Alectoris rufa x chukar* | 27/0/0 | 2.2 | - | - |
| *Alethe castanea* | 0/0/10 | - | - | 1.4 |
| *Alethe choloensis* | 0/0/2 | - | - | 0.3 |
| *Alethe diademata* | 0/0/2 | - | - | 0.7 |
| *Alethe fuelleborni* | 0/0/3 | - | - | 0.6 |
| *Alethe poliocephala* | 0/0/14 | - | - | 4.5 |
| *Alethe poliophrys* | 0/0/3 | - | - | 0.1 |
| *Alle alle* | 3/0/3 | 0.1 | - | 0.5 |
| *Amazona aestiva* | 0/0/2 | - | - | 0.8 |
| *Amazona ochrocephala* | 0/0/3 | - | - | 1.5 |
| *Ammodramus bairdii* | 2/0/0 | 0.0 | - | - |
| *Ammodramus caudacutus* | 3/0/0 | 0.0 | - | - |
| *Ammodramus maritimus* | 3/0/0 | 0.0 | - | - |
| *Ammomanes cincturus* | 0/2/2 | - | 0.0 | 0.0 |
| *Amphispiza belli* | 5/0/0 | 0.1 | - | - |
| *Amphispiza bilineata* | 6/0/0 | 0.2 | - | - |
| *Anas acuta* | 7/0/0 | 0.1 | - | - |
| *Anas americana* | 8/0/0 | 0.2 | - | - |
| *Anas aucklandica* | 0/0/2 | - | - | 1.3 |
| *Anas bahamensis* | 0/0/2 | - | - | 0.2 |
| *Anas clypeata* | 8/0/0 | 0.2 | - | - |
| *Anas crecca* | 8/0/0 | 0.0 | - | - |
| *Anas cyanoptera* | 2/0/2 | 0.0 | - | 0.2 |
| *Anas discors* | 8/0/0 | 0.0 | - | - |
| *Anas flavirostris* | 0/0/2 | - | - | 0.0 |
| *Anas platyrhynchos* | 8/4/2 | 0.6 | 3.1 | 0.6 |
| *Anas rubripes* | 8/0/0 | 0.1 | - | - |
| *Anas strepera* | 6/0/0 | 0.1 | - | - |
| *Andigena cucullata* | 0/0/2 | - | - | 0.0 |
| *Anhinga anhinga* | 3/0/0 | 0.3 | - | - |
| *Anisognathus flavinuchus* | 0/0/2 | - | - | 0.0 |
| *Anodorhynchus hyacinthinus* | 0/0/2 | - | - | 0.0 |
| *Anodorhynchus leari* | 0/0/2 | - | - | 0.4 |
| *Anomalopteryx didiformis* | 0/2/0 | - | 0.0 | - |
| *Anser albifrons* | 3/2/0 | 0.9 | 0.0 | - |
| *Anser cygnoides* | 0/0/4 | - | - | 1.4 |
| *Anseranas semipalmata* | 0/2/0 | - | 0.0 | - |
| *Anthracoceros albirostris* | 0/0/3 | - | - | 0.9 |
| *Anthracoceros malayanus* | 0/0/2 | - | - | 0.4 |
| *Anthus cinnamomeus* | 0/0/2 | - | - | 11.9 |
| *Anthus petrosus* | 0/2/0 | - | 0.0 | - |
| *Anthus pratensis* | 0/4/0 | - | 0.1 | - |
| *Anthus rubescens* | 2/0/0 | 0.0 | - | - |
| *Anthus spinoletta* | 6/3/0 | 0.7 | 0.1 | - |
| *Aphanotriccus audax* | 0/0/2 | - | - | 0.3 |
| *Aphelocephala leucopsis* | 0/2/0 | - | 0.0 | - |
| *Aphelocoma californica* | 8/0/0 | 0.8 | - | - |
| *Aphelocoma coerulescens* | 2/0/0 | 0.0 | - | - |
| *Aphelocoma insularis* | 2/0/0 | 0.0 | - | - |
| *Aphelocoma ultramarina* | 2/0/0 | 0.1 | - | - |
| *Aphriza virgata* | 2/0/0 | 0.0 | - | - |
| *Aptenodytes patagonicus* | 0/0/3 | - | - | 2.9 |
| *Apteryx haastii* | 0/2/0 | - | 0.0 | - |
| *Apus melba* | 0/0/4 | - | - | 5.0 |
| *Aquila audax* | 0/0/3 | - | - | 0.2 |
| *Aquila ayresii* | 0/0/2 | - | - | 0.2 |
| *Aquila chrysaetos* | 2/0/5 | 0.0 | - | 0.5 |
| *Aquila clanga* | 0/0/3 | - | - | 0.0 |
| *Aquila fasciatus* | 0/0/5 | - | - | 1.3 |
| *Aquila heliaca* | 0/0/3 | - | - | 0.2 |
| *Aquila kienerii* | 0/0/2 | - | - | 0.8 |
| *Aquila morphnoides* | 0/0/5 | - | - | 2.0 |
| *Aquila nipalensis* | 0/0/2 | - | - | 0.1 |
| *Aquila pennatus* | 0/0/4 | - | - | 0.2 |
| *Aquila pomarina* | 0/0/3 | - | - | 2.4 |
| *Aquila verreauxii* | 0/0/2 | - | - | 0.0 |
| *Aquila vindhiana* | 0/0/2 | - | - | 0.2 |
| *Aquila wahlbergi* | 0/0/2 | - | - | 0.2 |
| *Ara ararauna* | 0/0/2 | - | - | 0.6 |
| *Ara severus* | 0/0/2 | - | - | 0.4 |
| *Aramus guarauna* | 3/0/0 | 0.2 | - | - |
| *Aratinga aurea* | 0/0/2 | - | - | 0.1 |
| *Archilochus alexandri* | 2/0/0 | 0.1 | - | - |
| *Archilochus colubris* | 5/0/0 | 0.1 | - | - |
| *Ardea herodias* | 4/0/0 | 0.1 | - | - |
| *Ardeotis arabs* | 0/0/9 | - | - | 0.0 |
| *Ardeotis kori* | 0/0/3 | - | - | 0.7 |
| *Arenaria interpres* | 4/2/0 | 0.2 | 0.0 | - |
| *Asio flammeus* | 5/0/0 | 0.0 | - | - |
| *Asio otus* | 6/0/0 | 0.0 | - | - |
| *Atelornis crossleyi* | 0/0/2 | - | - | 0.3 |
| *Atelornis pittoides* | 0/0/5 | - | - | 0.6 |
| *Athene cunicularia* | 2/0/0 | 0.0 | - | - |
| *Athene noctua* | 0/0/5 | - | - | 3.3 |
| *Aulacorhynchus prasinus* | 0/0/2 | - | - | 0.0 |
| *Auriparus flaviceps* | 3/0/0 | 0.1 | - | - |
| *Aythya affinis* | 10/0/0 | 0.0 | - | - |
| *Aythya americana* | 10/2/0 | 0.1 | 0.0 | - |
| *Aythya collaris* | 8/0/0 | 0.1 | - | - |
| *Aythya fuligula* | 3/0/0 | 0.2 | - | - |
| *Aythya marila* | 9/0/0 | 0.0 | - | - |
| *Aythya valisineria* | 6/0/0 | 0.3 | - | - |
| *Baeolophus atricristatus* | 2/0/0 | 0.1 | - | - |
| *Baeolophus bicolor* | 2/0/2 | 0.3 | - | 0.0 |
| *Baeolophus inornatus* | 2/0/0 | 0.0 | - | - |
| *Baeolophus ridgwayi* | 2/0/0 | 0.1 | - | - |
| *Baillonius bailloni* | 0/0/3 | - | - | 1.7 |
| *Balaeniceps rex* | 0/2/0 | - | 0.0 | - |
| *Bartramia longicauda* | 3/0/0 | 0.1 | - | - |
| *Basileuterus flaveolus* | 2/0/0 | 0.3 | - | - |
| *Basileuterus fulvicauda* | 7/0/0 | 2.7 | - | - |
| *Basileuterus rivularis* | 4/0/0 | 3.4 | - | - |
| *Basileuterus rufifrons* | 0/0/2 | - | - | 4.4 |
| *Basileuterus tristriatus* | 0/0/3 | - | - | 3.1 |
| *Bombycilla cedrorum* | 3/0/4 | 0.1 | - | 0.1 |
| *Bombycilla garrulus* | 2/0/5 | 1.3 | - | 2.2 |
| *Bombycilla japonica* | 0/0/2 | - | - | 0.0 |
| *Bonasa umbellus* | 7/0/0 | 0.1 | - | - |
| *Botaurus lentiginosus* | 3/0/0 | 0.0 | - | - |
| *Brachyramphus brevirostris* | 7/0/5 | 0.6 | - | 1.2 |
| *Brachyramphus marmoratus* | 3/0/46 | 0.1 | - | 0.8 |
| *Branta bernicla* | 10/0/0 | 0.0 | - | - |
| *Branta canadensis* | 122/2/0 | 0.2 | 0.0 | - |
| *Branta hutchinsi* | 27/0/0 | 0.2 | - | - |
| *Brotogeris versicolurus* | 0/0/2 | - | - | 0.6 |
| *Bubo africanus* | 0/0/3 | - | - | 2.6 |
| *Bubo bubo* | 0/0/17 | - | - | 0.4 |
| *Bubo virginianus* | 6/0/3 | 1.1 | - | 0.4 |
| *Bubulcus ibis* | 3/0/0 | 0.0 | - | - |
| *Bucanetes githagineus* | 3/3/0 | 0.1 | 0.0 | - |
| *Bucephala albeola* | 8/0/0 | 0.1 | - | - |
| *Bucephala clangula* | 6/0/0 | 0.2 | - | - |
| *Bucephala islandica* | 11/0/0 | 0.0 | - | - |
| *Bulweria bulwerii* | 0/0/3 | - | - | 0.4 |
| *Buteo albicaudatus* | 2/0/0 | 0.3 | - | - |
| *Buteo buteo* | 0/3/2 | - | 0.0 | 1.6 |
| *Buteo jamaicensis* | 0/0/2 | - | - | 0.1 |
| *Buteo lineatus* | 2/0/0 | 0.1 | - | - |
| *Buteo platypterus* | 3/0/0 | 0.2 | - | - |
| *Buteo swainsoni* | 3/0/0 | 0.0 | - | - |
| *Buteogallus anthracinus* | 2/0/0 | 0.0 | - | - |
| *Buthraupis montana* | 0/0/2 | - | - | 0.1 |
| *Butorides virescens* | 2/0/0 | 0.9 | - | - |
| *Bycanistes brevis* | 0/0/6 | - | - | 0.3 |
| *Bycanistes bucinator* | 0/0/3 | - | - | 1.0 |
| *Cairina moschata* | 2/0/2 | 0.1 | - | 0.2 |
| *Calamospiza melanocorys* | 4/0/0 | 0.1 | - | - |
| *Calandrella rufescens* | 0/2/2 | - | 3.3 | 7.9 |
| *Calcarius lapponicus* | 3/0/41 | 0.6 | - | 2.6 |
| *Calcarius mccownii* | 4/0/0 | 0.3 | - | - |
| *Calcarius ornatus* | 7/0/0 | 0.3 | - | - |
| *Calidris alba* | 3/0/0 | 0.3 | - | - |
| *Calidris alpina* | 3/0/2 | 0.7 | - | 0.2 |
| *Calidris bairdii* | 2/0/0 | 0.0 | - | - |
| *Calidris canutus* | 2/0/0 | 0.0 | - | - |
| *Calidris fuscicollis* | 2/0/0 | 0.4 | - | - |
| *Calidris himantopus* | 2/0/0 | 0.1 | - | - |
| *Calidris maritima* | 0/0/4 | - | - | 0.1 |
| *Calidris mauri* | 4/0/0 | 0.1 | - | - |
| *Calidris melanotos* | 2/0/0 | 0.0 | - | - |
| *Calidris minutilla* | 3/0/0 | 0.4 | - | - |
| *Calidris ptilocnemis* | 0/0/58 | - | - | 0.1 |
| *Calidris pusilla* | 3/0/0 | 0.0 | - | - |
| *Callipepla gambelii* | 2/0/0 | 0.0 | - | - |
| *Callipepla squamata* | 2/0/0 | 0.0 | - | - |
| *Calochaetes coccineus* | 0/0/2 | - | - | 0.0 |
| *Calonectris diomedea* | 2/0/20 | 0.0 | - | 1.2 |
| *Calorhamphus fuliginosus* | 0/0/2 | - | - | 0.0 |
| *Calypte anna* | 4/0/0 | 0.0 | - | - |
| *Camarhynchus psittacula* | 0/2/0 | - | 0.0 | - |
| *Campephilus haematogaster* | 3/0/3 | 0.0 | - | 1.0 |
| *Camptostoma imberbe* | 2/0/0 | 0.0 | - | - |
| *Campylorhamphus trochilirostris* | 0/0/2 | - | - | 0.5 |
| *Campylorhynchus brunneicapillus* | 6/0/2 | 0.1 | - | 0.3 |
| *Campylorhynchus fasciatus* | 0/0/2 | - | - | 3.8 |
| *Campylorhynchus rufinucha* | 0/0/2 | - | - | 3.2 |
| *Campylorhynchus turdinus* | 0/0/2 | - | - | 2.9 |
| *Campylorhynchus yucatanicus* | 0/0/2 | - | - | 0.0 |
| *Campylorhynchus zonatus* | 0/0/4 | - | - | 5.1 |
| *Canachites canadensis* | 4/0/0 | 0.3 | - | - |
| *Capito auratus* | 0/0/20 | - | - | 4.4 |
| *Capito dayi* | 0/0/2 | - | - | 0.0 |
| *Capito maculicoronatus* | 0/0/2 | - | - | 0.3 |
| *Capito niger* | 0/0/2 | - | - | 7.2 |
| *Caprimulgus vociferus* | 4/0/0 | 0.2 | - | - |
| *Cardellina rubrifrons* | 0/0/2 | - | - | 0.0 |
| *Cardinalis cardinalis* | 8/0/0 | 0.2 | - | - |
| *Cardinalis cardinalis* | 0/0/2 | - | - | 8.7 |
| *Carduelis carduelis* | 3/3/2 | 0.2 | 1.7 | 4.1 |
| *Carduelis chloris* | 0/0/2 | - | - | 0.4 |
| *Carduelis flammea* | 3/0/0 | 1.2 | - | - |
| *Carduelis hornemanni* | 5/0/0 | 0.3 | - | - |
| *Carduelis lawrencei* | 2/0/0 | 0.0 | - | - |
| *Carduelis pinus* | 6/0/0 | 0.2 | - | - |
| *Carduelis tristis* | 8/0/0 | 0.2 | - | - |
| *Carpodacus cassinii* | 5/0/0 | 0.0 | - | - |
| *Carpodacus erythrinus* | 0/0/2 | - | - | 9.0 |
| *Carpodacus mexicanus* | 4/0/0 | 0.4 | - | - |
| *Carpodacus purpureus* | 6/0/0 | 0.3 | - | - |
| *Carpospiza brachydactyla* | 2/2/0 | 0.0 | 0.0 | - |
| *Casuarius casuarius* | 2/0/0 | 0.0 | - | - |
| *Catharacta antarctica* | 0/0/2 | - | - | 0.1 |
| *Catharacta chilensis* | 0/0/9 | - | - | 0.4 |
| *Catharacta hamiltoni* | 0/0/2 | - | - | 0.0 |
| *Catharacta lonnbergi* | 0/0/7 | - | - | 0.3 |
| *Catharacta maccormicki* | 0/0/7 | - | - | 0.0 |
| *Catharacta skua* | 2/0/5 | 0.0 | - | 0.1 |
| *Cathartes aura* | 4/2/0 | 0.2 | 0.0 | - |
| *Cathartes burrovianus* | 0/0/3 | - | - | 3.3 |
| *Cathartes melambrotus* | 0/0/2 | - | - | 0.1 |
| *Catharus aurantiirostris* | 0/0/2 | - | - | 7.5 |
| *Catharus bicknelli* | 11/0/0 | 0.0 | - | - |
| *Catharus frantzii* | 0/0/2 | - | - | 0.5 |
| *Catharus fuscescens* | 5/0/0 | 0.1 | - | - |
| *Catharus fuscescens* | 0/0/2 | - | - | 0.0 |
| *Catharus guttatus* | 15/0/4 | 1.4 | - | 2.0 |
| *Catharus mexicanus* | 0/0/2 | - | - | 0.5 |
| *Catharus minimus* | 3/0/2 | 0.1 | - | 0.4 |
| *Catharus occidentalis* | 0/0/2 | - | - | 0.6 |
| *Catharus ustulatus* | 7/0/2 | 0.0 | - | 1.2 |
| *Catherpes mexicanus* | 2/0/0 | 0.1 | - | - |
| *Centrocercus minimus* | 2/0/0 | 0.0 | - | - |
| *Centrocercus urophasianus* | 5/0/0 | 0.0 | - | - |
| *Cepphus columba* | 2/0/0 | 0.0 | - | - |
| *Cepphus grylle* | 4/0/2 | 0.1 | - | 1.2 |
| *Cerorhinca monocerata* | 4/0/0 | 0.0 | - | - |
| *Certhia americana* | 3/0/0 | 0.1 | - | - |
| *Certhia familiaris* | 2/0/0 | 0.3 | - | - |
| *Certhidea olivacea* | 0/3/0 | - | 0.3 | - |
| *Cettia cetti* | 0/0/5 | - | - | 2.2 |
| *Cettia diphone* | 0/0/7 | - | - | 1.1 |
| *Chaetura pelagica* | 2/0/2 | 0.0 | - | 0.4 |
| *Chaetura vauxi* | 8/0/0 | 0.1 | - | - |
| *Chalcophaps indica* | 0/0/2 | - | - | 0.4 |
| *Chamaea fasciata* | 2/0/61 | 0.3 | - | 0.3 |
| *Charadrius alexandrinus* | 3/0/0 | 0.1 | - | - |
| *Charadrius hiaticula* | 2/0/0 | 0.4 | - | - |
| *Charadrius melodus* | 6/0/0 | 0.0 | - | - |
| *Charadrius montanus* | 2/0/0 | 0.0 | - | - |
| *Charadrius semipalmatus* | 5/0/0 | 0.0 | - | - |
| *Charadrius vociferus* | 3/0/0 | 0.1 | - | - |
| *Charadrius wilsonia* | 2/0/0 | 0.0 | - | - |
| *Chen caerulescens* | 4/0/0 | 0.1 | - | - |
| *Chen rossii* | 2/0/0 | 0.1 | - | - |
| *Chlamydotis macqueenii* | 0/0/17 | - | - | 0.2 |
| *Chlamydotis undulata* | 0/0/11 | - | - | 0.4 |
| *Chlidonias niger* | 2/0/0 | 0.0 | - | - |
| *Chloroceryle americana* | 4/0/0 | 0.2 | - | - |
| *Chlorochrysa calliparaea* | 0/0/2 | - | - | 0.0 |
| *Chlorornis riefferii* | 0/0/2 | - | - | 0.0 |
| *Chondestes grammacus* | 2/0/0 | 1.6 | - | - |
| *Chordeiles minor* | 2/0/0 | 1.2 | - | - |
| *Chrysolophus amherstiae* | 0/0/3 | - | - | 1.3 |
| *Chrysolophus pictus* | 0/0/3 | - | - | 1.3 |
| *Cichlherminia lherminieri* | 0/0/2 | - | - | 0.0 |
| *Cichlopsis leucogenys* | 0/0/2 | - | - | 0.1 |
| *Ciconia boyciana* | 0/2/0 | - | 0.0 | - |
| *Ciconia ciconia* | 0/2/2 | - | 0.0 | 0.4 |
| *Ciconia episcopus* | 0/0/2 | - | - | 0.9 |
| *Ciconia nigra* | 0/2/0 | - | 0.0 | - |
| *Cinclocerthia ruficauda* | 0/2/0 | - | 0.4 | - |
| *Cinclus mexicanus* | 9/0/2 | 0.2 | - | 0.0 |
| *Circaetus gallicus* | 0/0/2 | - | - | 6.5 |
| *Circus cyaneus* | 4/0/0 | 0.1 | - | - |
| *Cissopis leveriana* | 0/0/2 | - | - | 0.0 |
| *Cisticola juncidis* | 0/0/3 | - | - | 1.5 |
| *Cistothorus palustris* | 5/0/0 | 4.3 | - | - |
| *Cistothorus platensis* | 2/0/0 | 0.0 | - | - |
| *Clangula hyemalis* | 8/0/0 | 0.1 | - | - |
| *Coccyzus americanus* | 0/2/11 | - | 0.0 | 1.2 |
| *Coccyzus erythropthalmus* | 4/0/2 | 0.2 | - | 7.1 |
| *Coereba flaveola* | 0/0/5 | - | - | 4.1 |
| *Colaptes auratus* | 16/0/3 | 0.3 | - | 2.0 |
| *Colaptes rupicola* | 0/0/2 | - | - | 2.1 |
| *Colinus virginianus* | 3/0/0 | 0.4 | - | - |
| *Colius striatus* | 0/0/2 | - | - | 9.6 |
| *Collocalia esculenta* | 0/0/21 | - | - | 4.7 |
| *Collocalia linchi* | 0/0/6 | - | - | 2.5 |
| *Columba livia* | 2/2/0 | 0.0 | 0.0 | - |
| *Columba palumbus* | 0/0/2 | - | - | 0.1 |
| *Columbina inca* | 6/0/0 | 0.0 | - | - |
| *Columbina passerina* | 8/0/0 | 0.1 | - | - |
| *Conirostrum bicolor* | 0/0/2 | - | - | 0.7 |
| *Contopus cooperi* | 0/0/2 | - | - | 0.2 |
| *Contopus sordidulus* | 6/0/2 | 0.9 | - | 0.3 |
| *Contopus sordidulus* | 0/0/2 | - | - | 0.3 |
| *Contopus virens* | 0/0/2 | - | - | 0.2 |
| *Copsychus albospecularis* | 0/0/2 | - | - | 0.3 |
| *Coragyps atratus* | 5/0/0 | 0.0 | - | - |
| *Corvus brachyrhynchos* | 3/0/0 | 0.4 | - | - |
| *Corvus caurinus* | 3/0/0 | 0.2 | - | - |
| *Corvus corax* | 6/0/42 | 2.4 | - | 0.3 |
| *Corvus cryptoleucus* | 2/0/0 | 0.1 | - | - |
| *Corvus frugilegus* | 2/2/0 | 0.0 | 0.0 | - |
| *Corvus macrorhynchos* | 0/0/38 | - | - | 0.4 |
| *Corvus ossifragus* | 2/0/0 | 0.1 | - | - |
| *Corythaixoides concolor* | 0/0/2 | - | - | 4.0 |
| *Cossypha archeri* | 0/0/2 | - | - | 2.8 |
| *Cossypha dichroa* | 0/0/3 | - | - | 6.1 |
| *Cossypha polioptera* | 0/0/2 | - | - | 4.5 |
| *Coturnix chinensis* | 0/2/0 | - | 0.0 | - |
| *Coturnix coturnix* | 0/0/7 | - | - | 0.6 |
| *Coturnix japonica* | 0/2/0 | - | 0.0 | - |
| *Crax rubra* | 0/0/2 | - | - | 0.8 |
| *Criniger ochraceus* | 0/2/0 | - | 3.3 | - |
| *Criniger phaeocephalus* | 0/7/0 | - | 1.1 | - |
| *Crotophaga ani* | 4/0/2 | 0.1 | - | - |
| *Cuculus pallidus* | 0/2/0 | - | 0.0 | - |
| *Cyanocitta cristata* | 9/0/0 | 0.0 | - | - |
| *Cyanocitta stelleri* | 6/0/0 | 0.0 | - | - |
| *Cyanocorax yncas* | 2/0/0 | 0.1 | - | - |
| *Cyanopsitta spixii* | 0/0/2 | - | - | 0.1 |
| *Cyanoramphus auriceps* | 0/0/3 | - | - | 4.8 |
| *Cyanoramphus malherbi* | 0/0/2 | - | - | 0.0 |
| *Cyanoramphus novaezelandiae* | 0/0/2 | - | - | 0.3 |
| *Cyclarhis gujanensis* | 0/0/2 | - | - | 6.7 |
| *Cyclorrhynchus psittacula* | 2/0/0 | 0.3 | - | - |
| *Cygnus buccinator* | 2/0/0 | 0.0 | - | - |
| *Cygnus columbianus* | 2/2/2 | 0.0 | 0.0 | 0.0 |
| *Cygnus olor* | 2/0/0 | 0.0 | - | - |
| *Dasyornis broadbenti* | 0/2/0 | - | 0.2 | - |
| *Delichon urbicum* | 0/2/0 | - | 1.5 | - |
| *Delothraupis castaneoventris* | 0/2/0 | - | 0.0 | - |
| *Dendragapus obscurus* | 6/0/0 | 0.7 | - | - |
| *Dendrexetastes rufigula* | 0/0/2 | - | - | 1.5 |
| *Dendrocolaptes certhia* | 0/0/2 | - | - | 1.6 |
| *Dendrocopos major* | 2/0/2 | 0.1 | - | 0.6 |
| *Dendrocopos kizuki* | 2/0/2 | 0.1 | - | 0.1 |
| *Dendrocopos leucotos* | 2/0/2 | 0.0 | - | 0.2 |
| *Dendrocopos minor* | 2/0/2 | 0.0 | - | 0.0 |
| *Dendroica adelaidae* | 0/0/2 | - | - | 4.0 |
| *Dendroica caerulescens* | 5/0/0 | 0.0 | - | - |
| *Dendroica castanea* | 5/0/0 | 0.1 | - | - |
| *Dendroica cerulea* | 2/0/0 | 0.1 | - | - |
| *Dendroica coronata* | 6/0/0 | 0.3 | - | - |
| *Dendroica discolor* | 6/0/2 | 0.3 | - | 0.8 |
| *Dendroica dominica* | 3/0/0 | 0.6 | - | - |
| *Dendroica fusca* | 5/0/0 | 0.0 | - | - |
| *Dendroica graciae* | 4/0/0 | 0.1 | - | - |
| *Dendroica kirtlandii* | 2/0/0 | 0.0 | - | - |
| *Dendroica magnolia* | 4/0/0 | 0.2 | - | - |
| *Dendroica nigrescens* | 8/0/0 | 0.2 | - | - |
| *Dendroica occidentalis* | 5/0/0 | 0.2 | - | - |
| *Dendroica palmarum* | 8/0/0 | 0.2 | - | - |
| *Dendroica pensylvanica* | 4/0/2 | 0.2 | - | 0.0 |
| *Dendroica petechia* | 5/0/3 | 0.5 | - | 1.5 |
| *Dendroica pinus* | 5/0/4 | 0.2 | - | 0.3 |
| *Dendroica striata* | 6/0/0 | 0.1 | - | - |
| *Dendroica tigrina* | 4/0/2 | 0.7 | - | 0.1 |
| *Dendroica townsendi* | 6/0/0 | 0.2 | - | - |
| *Dendroica virens* | 4/0/0 | 0.3 | - | - |
| *Dendroica vitellina* | 0/0/4 | - | - | 0.4 |
| *Dendropicos fuscescens* | 2/0/0 | 1.1 | - | - |
| *Dendropicos fuscescens* | 0/0/2 | - | - | 0.0 |
| *Dendropicos griseocephalus* | 2/0/2 | 0.0 | - | 0.5 |
| *Dinornis giganteus* | 0/2/0 | - | 0.0 | - |
| *Diomedea epomophora* | 0/0/2 | - | - | 0.1 |
| *Diomedea exulans* | 0/0/2 | - | - | 10.8 |
| *Dolichonyx oryzivorus* | 6/0/0 | 0.0 | - | - |
| *Dromaius novaehollandiae* | 0/4/0 | - | 0.3 | - |
| *Dryocopus pileatus* | 7/0/2 | 0.8 | - | 0.9 |
| *Dubusia taeniata* | 0/0/2 | - | - | 0.0 |
| *Dumetella carolinensis* | 6/2/0 | 0.2 | 0.0 | - |
| *Egretta caerulea* | 2/0/0 | 0.0 | - | - |
| *Egretta tricolor* | 3/0/0 | 0.0 | - | - |
| *Elanus leucurus* | 2/0/0 | 0.0 | - | - |
| *Emberiza bruniceps* | 3/3/0 | 0.3 | 0.4 | - |
| *Emberiza caesia* | 0/2/0 | - | 0.0 | - |
| *Emberiza cia* | 2/2/0 | 0.3 | 0.4 | - |
| *Emberiza cioides* | 0/0/4 | - | - | 0.1 |
| *Emberiza citrinella* | 0/0/2 | - | - | 0.1 |
| *Emberiza elegans* | 0/0/3 | - | - | 0.5 |
| *Emberiza fucata* | 0/0/2 | - | - | 0.4 |
| *Emberiza hortulana* | 0/3/0 | - | 0.1 | - |
| *Emberiza rustica* | 0/0/3 | - | - | 9.9 |
| *Emberiza rutila* | 0/0/2 | - | - | 0.3 |
| *Emberiza tristrami* | 0/0/2 | - | - | 0.2 |
| *Emeus crassus* | 0/3/0 | - | 0.0 | - |
| *Empidonax albigularis* | 0/0/2 | - | - | 0.2 |
| *Empidonax alnorum* | 6/0/3 | 0.0 | - | 0.1 |
| *Empidonax atriceps* | 0/0/2 | - | - | 0.0 |
| *Empidonax difficilis* | 5/0/4 | 0.0 | - | 3.9 |
| *Empidonax flavescens* | 0/0/3 | - | - | 0.9 |
| *Empidonax flaviventris* | 6/0/2 | 0.4 | - | 0.1 |
| *Empidonax fulvifrons* | 2/0/2 | 0.0 | - | 0.5 |
| *Empidonax hammondii* | 5/0/2 | 0.1 | - | 0.5 |
| *Empidonax minimus* | 4/0/4 | 0.3 | - | 1.2 |
| *Empidonax oberholseri* | 0/0/2 | - | - | 0.3 |
| *Empidonax occidentalis* | 0/0/4 | - | - | 0.2 |
| *Empidonax traillii* | 4/0/42 | 0.4 | - | 0.3 |
| *Empidonax virescens* | 0/0/2 | - | - | 0.2 |
| *Empidonax wrightii* | 0/0/3 | - | - | 0.3 |
| *Ephippiorhynchus asiaticus* | 0/0/2 | - | - | 2.1 |
| *Eremophila alpestris* | 4/0/0 | 2.2 | - | - |
| *Eremophila bilopha* | 0/2/0 | - | 1.0 | - |
| *Eudocimus albus* | 6/0/0 | 0.0 | - | - |
| *Eudyptes chrysocome* | 0/0/3 | - | - | 1.1 |
| *Eudyptes chrysolophus* | 0/0/2 | - | - | 0.3 |
| *Eudyptula minor* | 0/3/0 | - | 0.1 | - |
| *Eugenes fulgens* | 2/0/0 | 0.4 | - | - |
| *Euphagus carolinus* | 3/0/0 | 0.0 | - | - |
| *Euphagus cyanocephalus* | 6/0/0 | 0.1 | - | - |
| *Eupodotis rueppellii* | 0/0/4 | - | - | 0.2 |
| *Eupodotis senegalensis* | 0/0/4 | - | - | 0.4 |
| *Falco columbarius* | 4/0/0 | 0.0 | - | - |
| *Falco peregrinus* | 3/2/2 | 0.2 | 0.0 | 0.9 |
| *Falco rusticolus* | 2/0/0 | 0.0 | - | - |
| *Falco sparverius* | 5/0/0 | 0.3 | - | - |
| *Falco tinnunculus* | 0/0/9 | - | - | 0.7 |
| *Ficedula mugimaki* | 0/0/2 | - | - | 0.6 |
| *Ficedula parva* | 0/0/5 | - | - | 0.4 |
| *Fratercula arctica* | 6/0/3 | 0.1 | - | 0.1 |
| *Fratercula cirrhata* | 2/0/0 | 0.0 | - | - |
| *Fratercula corniculata* | 2/0/0 | 0.0 | - | - |
| *Fregata magnificens* | 2/2/0 | 0.0 | 0.0 | - |
| *Fringilla coelebs* | 0/0/3 | - | - | 0.1 |
| *Fringilla montifringilla* | 2/0/5 | 0.1 | - | 6.9 |
| *Fulica americana* | 5/0/0 | 0.1 | - | - |
| *Fulmarus glacialis* | 4/0/4 | 1.6 | - | 2.4 |
| *Fulmarus glacialoides* | 0/0/4 | - | - | 0.7 |
| *Galerida cristata* | 0/4/4 | - | 0.0 | 1.6 |
| *Galerida theklae* | 0/0/4 | - | - | 0.1 |
| *Gallinago delicata* | 4/0/0 | 0.1 | - | - |
| *Gallinula chloropus* | 4/0/0 | 0.0 | - | - |
| *Gallus gallus* | 4/4/4 | 0.2 | 0.1 | 0.4 |
| *Gallus lafayettei* | 0/0/4 | - | - | 0.9 |
| *Gallus sonneratii* | 4/4/4 | 3.9 | 2.3 | 3.3 |
| *Garrulax canorus* | 0/0/4 | - | - | 1.7 |
| *Garrulax chinensis* | 0/0/4 | - | - | 1.3 |
| *Garrulax poecilorhynchus* | 0/0/4 | - | - | 2.0 |
| *Gavia adamsii* | 4/0/0 | 0.0 | - | - |
| *Gavia immer* | 4/4/4 | 0.0 | 0.1 | 0.5 |
| *Gavia pacifica* | 4/0/0 | 0.0 | - | - |
| *Gavia stellata* | 4/4/0 | 0.0 | 0.0 | - |
| *Geopelia cuneata* | 0/0/4 | - | - | 3.8 |
| *Geothlypis trichas* | 4/0/4 | 0.5 | - | 0.2 |
| *Geronticus eremita* | 0/2/0 | - | 0.0 | - |
| *Glaucidium brasilianum* | 4/0/0 | 0.0 | - | - |
| *Glaucidium hardyi* | 0/0/4 | - | - | 0.1 |
| *Glaucidium passerinum* | 0/0/4 | - | - | 0.2 |
| *Glyphorynchus spirurus* | 0/0/4 | - | - | 6.8 |
| *Grallaria squamigera* | 0/0/4 | - | - | 9.4 |
| *Grus americana* | 4/0/0 | 0.0 | - | - |
| *Grus canadensis* | 0/0/4 | - | - | 0.7 |
| *Guaruba guarouba* | 0/0/4 | - | - | 0.1 |
| *Gymnogyps californianus* | 0/4/0 | - | 0.0 | - |
| *Gymnorhinus cyanocephalus* | 6/0/2 | 0.0 | - | 0.7 |
| *Gypaetus barbatus* | 0/0/3 | - | - | 4.8 |
| *Gyps africanus* | 0/0/2 | - | - | 0.2 |
| *Gyps bengalensis* | 0/0/2 | - | - | 0.2 |
| *Gyps coprotheres* | 0/0/2 | - | - | 0.6 |
| *Gyps fulvus* | 0/0/2 | - | - | 0.0 |
| *Haematopus ater* | 0/2/0 | - | 0.0 | - |
| *Haematopus bachmani* | 4/0/0 | 0.0 | - | - |
| *Haematopus palliatus* | 2/0/0 | 0.0 | - | - |
| *Haliaeetus albicilla* | 0/0/2 | - | - | 0.0 |
| *Haliaeetus leucocephalus* | 4/0/2 | 0.0 | - | 0.0 |
| *Haliaeetus leucogaster* | 0/0/2 | - | - | 0.2 |
| *Haliaeetus pelagicus* | 0/0/2 | - | - | 0.0 |
| *Haliaeetus vocifer* | 0/0/2 | - | - | 0.2 |
| *Harpagornis moorei* | 0/0/2 | - | - | 0.3 |
| *Harpia harpyja* | 0/0/2 | - | - | 0.1 |
| *Helmitheros vermivorus* | 3/0/2 | 0.4 | - | 0.2 |
| *Hemispingus atropileus* | 0/0/2 | - | - | 0.0 |
| *Hesperiphona vespertina* | 2/0/0 | 0.3 | - | - |
| *Himantopus mexicanus* | 3/0/0 | 0.0 | - | - |
| *Hippolais caligata* | 0/0/3 | - | - | 5.1 |
| *Hippolais icterina* | 4/4/2 | 1.5 | 0.2 | 0.3 |
| *Hippolais pallida* | 0/0/3 | - | - | 7.3 |
| *Hippolais polyglotta* | 0/2/0 | - | 0.4 | - |
| *Hirundo rustica* | 3/0/0 | 0.0 | - | - |
| *Histrionicus histrionicus* | 3/0/0 | 0.1 | - | - |
| *Hydrochous gigas* | 0/0/2 | - | - | 0.9 |
| *Hylexetastes perrotii* | 0/0/2 | - | - | 0.0 |
| *Hylocichla mustelina* | 4/0/2 | 1.1 | - | 1.8 |
| *Hylophilus ochraceiceps* | 0/0/2 | - | - | 0.6 |
| *Hypsipetes amaurotis* | 0/0/4 | - | - | 0.3 |
| *Hypsipetes madagascariensis* | 0/2/0 | - | 0.9 | - |
| *Icteria virens* | 2/0/2 | 0.3 | - | 1.0 |
| *Icterus bullockii* | 5/0/0 | 0.0 | - | - |
| *Icterus cucullatus* | 4/0/0 | 0.0 | - | - |
| *Icterus galbula* | 5/0/0 | 0.1 | - | - |
| *Icterus gularis* | 3/0/0 | 0.6 | - | - |
| *Iridosornis analis* | 0/0/2 | - | - | 0.0 |
| *Ixobrychus exilis* | 2/0/0 | 0.1 | - | - |
| *Ixoreus naevius* | 2/0/0 | 0.0 | - | - |
| *Jabiru mycteria* | 0/0/2 | - | - | 0.6 |
| *Jacana spinosa* | 2/0/0 | 0.0 | - | - |
| *Junco hyemalis* | 21/0/0 | 0.1 | - | - |
| *Junco phaeonotus* | 3/0/0 | 0.0 | - | - |
| *Lagopus lagopus* | 5/0/0 | 1.0 | - | - |
| *Lagopus leucurus* | 5/0/0 | 0.0 | - | - |
| *Lagopus mutus* | 20/0/42 | 0.1 | - | 0.1 |
| *Lampornis amethystinus* | 0/0/12 | - | - | 0.8 |
| *Lampornis calolaemus* | 0/0/4 | - | - | 0.4 |
| *Lampornis castaneoventris* | 0/0/5 | - | - | 0.5 |
| *Lampornis hemileucus* | 0/0/3 | - | - | 0.3 |
| *Lampornis sybillae* | 0/0/2 | - | - | 0.2 |
| *Lamprolaima rhami* | 0/0/2 | - | - | 0.0 |
| *Lanius bucephalus* | 0/0/4 | - | - | 0.2 |
| *Lanius collurio* | 0/3/0 | - | 1.0 | - |
| *Lanius cristatus* | 0/2/0 | - | 1.1 | - |
| *Lanius excubitor* | 3/0/0 | 1.7 | - | - |
| *Lanius ludovicianus* | 5/0/2 | 1.1 | - | 0.9 |
| *Lanius schach* | 2/2/0 | 2.5 | 0.2 | - |
| *Lanius tephronothus* | 0/2/0 | - | 0.6 | - |
| *Larus cachinnans* | 0/0/16 | - | - | 0.7 |
| *Larus argentatus* | 8/2/22 | 0.1 | 0.2 | 0.6 |
| *Larus californicus* | 5/0/16 | 0.0 | - | 0.7 |
| *Larus canus* | 4/0/0 | 0.3 | - | - |
| *Larus delawarensis* | 3/0/0 | 0.2 | - | - |
| *Larus dominicanus* | 0/2/0 | - | 0.0 | - |
| *Larus fuscus* | 5/0/15 | 0.0 | - | 0.4 |
| *Larus glaucescens* | 4/0/0 | 0.0 | - | - |
| *Larus glaucoides* | 0/0/2 | - | - | 0.1 |
| *Larus heermanni* | 3/0/0 | 0.3 | - | - |
| *Larus hyperboreus* | 4/0/6 | 0.1 | - | 0.9 |
| *Larus marinus* | 2/0/3 | 0.0 | - | 0.3 |
| *Larus michahellis* | 0/0/5 | - | - | 0.2 |
| *Larus occidentalis* | 4/0/0 | 0.1 | - | - |
| *Larus philadelphia* | 4/0/0 | 0.0 | - | - |
| *Larus pipixcan* | 4/0/2 | 0.2 | - | 0.0 |
| *Larus ridibundus* | 5/0/0 | 0.1 | - | - |
| *Larus thayeri* | 2/0/0 | 0.0 | - | - |
| *Lathrotriccus euleri* | 0/0/2 | - | - | 0.5 |
| *Lepidocolaptes angustirostris* | 0/0/2 | - | - | 0.1 |
| *Lepidocolaptes fuscus* | 0/0/2 | - | - | 1.8 |
| *Leptoptilos crumeniferus* | 0/0/2 | - | - | 2.7 |
| *Leptotila megalura* | 0/0/2 | - | - | 0.0 |
| *Leptotila verreauxi* | 3/0/3 | 0.5 | - | 3.5 |
| *Leucosarcia melanoleuca* | 0/0/2 | - | - | 0.3 |
| *Leucosticte tephrocotis* | 3/0/39 | 0.0 | - | 0.1 |
| *Limnodromus griseus* | 6/0/0 | 0.0 | - | - |
| *Limnodromus scolopaceus* | 2/0/0 | 0.0 | - | - |
| *Limnothlypis swainsonii* | 2/0/2 | 0.0 | - | 0.2 |
| *Limosa fedoa* | 3/0/2 | 0.0 | - | 0.0 |
| *Limosa haemastica* | 2/0/2 | 0.1 | - | 0.0 |
| *Limosa lapponica* | 2/0/3 | 0.1 | - | 0.2 |
| *Locustella fluviatilis* | 0/0/2 | - | - | 0.5 |
| *Locustella lanceolata* | 0/0/2 | - | - | 0.2 |
| *Locustella pleskei* | 0/0/7 | - | - | 3.4 |
| *Lophaetus occipitalis* | 0/0/2 | - | - | 0.0 |
| *Lophodytes cucullatus* | 8/0/0 | 0.1 | - | - |
| *Lophorina superba* | 0/0/2 | - | - | 1.2 |
| *Lophotis ruficrista* | 0/0/2 | - | - | 0.6 |
| *Lophura edwardsi* | 0/0/2 | - | - | 0.1 |
| *Lophura swinhoii* | 0/0/2 | - | - | 0.0 |
| *Loxia curvirostra* | 3/0/0 | 0.2 | - | - |
| *Loxia leucoptera* | 2/0/0 | 0.1 | - | - |
| *Luscinia cyane* | 0/0/2 | - | - | 2.1 |
| *Luscinia svecica* | 3/0/2 | 0.3 | - | 0.5 |
| *Macronectes giganteus* | 0/0/2 | - | - | 1.0 |
| *Macropygia phasianella* | 0/0/2 | - | - | 0.0 |
| *Magaceryle alcyon* | 2/0/0 | 0.0 | - | - |
| *Megaceryle torquata* | 3/0/0 | 0.0 | - | - |
| *Megalaima haemacephala* | 0/0/2 | - | - | 17.9 |
| *Megalurus pryeri* | 0/0/6 | - | - | 0.5 |
| *Megascops asio* | 8/0/0 | 0.1 | - | - |
| *Megascops kennicottii* | 8/0/0 | 1.5 | - | - |
| *Melanerpes carolinus* | 7/0/3 | 0.1 | - | 2.2 |
| *Melanerpes erythrocephalus* | 3/0/0 | 0.3 | - | - |
| *Melanerpes formicivorus* | 10/0/0 | 0.2 | - | - |
| *Melanerpes lewis* | 5/0/0 | 0.0 | - | - |
| *Melanerpes uropygialis* | 0/0/2 | - | - | 0.1 |
| *Melanitta fusca* | 3/0/0 | 0.1 | - | - |
| *Melanitta nigra* | 6/0/0 | 0.0 | - | - |
| *Melanochlora sultanea* | 0/0/2 | - | - | 0.9 |
| *Meleagris gallopavo* | 3/0/0 | 0.1 | - | - |
| *Melipotes fumigatus* | 0/0/2 | - | - | 3.7 |
| *Melopsittacus undulatus* | 4/2/0 | 0.2 | 0.0 | - |
| *Melospiza georgiana* | 4/0/0 | 0.1 | - | - |
| *Melospiza lincolnii* | 7/0/0 | 0.1 | - | - |
| *Melospiza melodia* | 5/0/64 | 0.8 | - | 0.2 |
| *Menura novaehollandiae* | 0/2/0 | - | 0.0 | - |
| *Mergus merganser* | 9/0/0 | 0.0 | - | - |
| *Mergus serrator* | 6/0/0 | 0.0 | - | - |
| *Micrathene whitneyi* | 2/0/0 | 0.1 | - | - |
| *Microligea palustris* | 0/0/3 | - | - | 0.2 |
| *Milvus migrans* | 0/0/19 | - | - | 1.0 |
| *Milvus milvus* | 0/0/13 | - | - | 0.0 |
| *Mirafra javanica* | 0/0/2 | - | - | 1.8 |
| *Mitrephanes olivaceus* | 0/0/2 | - | - | 6.2 |
| *Mniotilta varia* | 6/0/2 | 0.1 | - | 0.6 |
| *Molothrus aeneus* | 7/0/0 | 0.0 | - | - |
| *Molothrus ater* | 6/0/0 | 0.4 | - | - |
| *Molothrus bonariensis* | 2/0/0 | 0.0 | - | - |
| *Monticola sharpei* | 0/0/6 | - | - | 0.9 |
| *Morphnus guianensis* | 0/0/2 | - | - | 0.8 |
| *Morus bassanus* | 4/0/0 | 0.0 | - | - |
| *Motacilla aguimp* | 0/0/2 | - | - | 5.0 |
| *Motacilla alba* | 4/0/7 | 0.5 | - | 2.4 |
| *Motacilla capensis* | 0/0/2 | - | - | 6.0 |
| *Motacilla cinerea* | 0/0/2 | - | - | 1.1 |
| *Motacilla citreola* | 0/0/2 | - | - | 0.6 |
| *Motacilla clara* | 0/0/3 | - | - | 3.7 |
| *Motacilla flava* | 3/0/11 | 0.3 | - | 2.9 |
| *Motacilla flaviventris* | 0/0/2 | - | - | 1.4 |
| *Motacilla lugens* | 0/0/2 | - | - | 0.2 |
| *Muscicapa caerulescens* | 0/0/2 | - | - | 2.6 |
| *Musophaga violacea* | 0/0/2 | - | - | 3.4 |
| *Myadestes townsendi* | 7/0/2 | 0.1 | - | 0.0 |
| *Myadestes unicolor* | 0/0/2 | - | - | 0.1 |
| *Mycteria americana* | 2/0/2 | 0.0 | - | 0.2 |
| *Myiarchus cinerascens* | 5/0/0 | 0.1 | - | - |
| *Myiarchus crinitus* | 2/0/0 | 0.0 | - | - |
| *Myiarchus tuberculifer* | 5/0/0 | 0.1 | - | - |
| *Myiarchus tyrannulus* | 4/0/0 | 0.1 | - | - |
| *Myioborus albifacies* | 0/0/5 | - | - | 0.2 |
| *Myioborus albifrons* | 0/0/4 | - | - | 0.3 |
| *Myioborus brunniceps* | 0/0/5 | - | - | 3.0 |
| *Myioborus castaneocapillus* | 0/0/6 | - | - | 1.7 |
| *Myioborus flavivertex* | 0/0/4 | - | - | 0.1 |
| *Myioborus melanocephalus* | 0/0/7 | - | - | 0.7 |
| *Myioborus miniatus* | 0/0/14 | - | - | 2.2 |
| *Myioborus ornatus* | 0/0/6 | - | - | 0.4 |
| *Myioborus pictus* | 0/0/3 | - | - | 0.1 |
| *Myioborus torquatus* | 0/0/2 | - | - | 0.1 |
| *Myiodynastes luteiventris* | 3/0/0 | 0.1 | - | - |
| *Myiopsitta monachus* | 3/0/2 | 0.3 | - | 0.4 |
| *Nasica longirostris* | 0/0/2 | - | - | 0.6 |
| *Neophron percnopterus* | 0/2/3 | - | 0.0 | 6.1 |
| *Neothraupis fasciata* | 0/0/2 | - | - | 0.0 |
| *Ninox scutulata* | 0/0/2 | - | - | 0.9 |
| *Nomonyx dominicus* | 0/0/2 | - | - | 0.0 |
| *Nucifraga columbiana* | 8/0/0 | 0.1 | - | - |
| *Numenius americanus* | 2/0/0 | 0.1 | - | - |
| *Numenius phaeopus* | 2/0/3 | 0.3 | - | 3.3 |
| *Nyctea scandiaca* | 2/0/2 | 0.3 | - | 0.1 |
| *Nyctidromus albicollis* | 10/0/0 | 0.2 | - | - |
| *Oceanites oceanicus* | 2/0/0 | 0.0 | - | - |
| *Oceanodroma furcata* | 2/0/0 | 0.3 | - | - |
| *Oceanodroma leucorhoa* | 4/0/0 | 0.5 | - | - |
| *Oenanthe alboniger* | 3/3/0 | 0.6 | 0.0 | - |
| *Oenanthe deserti* | 4/4/0 | 0.5 | 1.5 | - |
| *Oenanthe finschii* | 3/3/0 | 0.0 | 0.0 | - |
| *Oenanthe hispanica* | 0/5/0 | - | 1.4 | - |
| *Oenanthe isabellina* | 6/6/0 | 0.2 | 0.0 | - |
| *Oenanthe lugens* | 3/4/0 | 0.1 | 0.0 | - |
| *Oenanthe oenanthe* | 7/5/0 | 0.6 | 0.4 | - |
| *Oenanthe pleschanka* | 2/2/0 | 0.3 | 0.4 | - |
| *Opisthocomus hoazin* | 3/2/4 | 0.2 | 0.0 | 4.0 |
| *Oporornis agilis* | 2/0/0 | 0.3 | - | - |
| *Oporornis formosus* | 2/0/2 | 0.3 | - | 0.2 |
| *Oporornis philadelphia* | 4/0/0 | 0.5 | - | - |
| *Oporornis tolmiei* | 3/0/0 | 0.2 | - | - |
| *Oreortyx pictus* | 4/0/0 | 0.1 | - | - |
| *Oreoscoptes montanus* | 6/0/0 | 0.1 | - | - |
| *Oroaetus isidori* | 0/0/2 | - | - | 0.4 |
| *Orthopsittaca manilata* | 0/0/2 | - | - | 0.1 |
| *Otis tarda* | 0/0/7 | - | - | 0.5 |
| *Otus bakkamoena* | 0/0/3 | - | - | 0.0 |
| *Otus flammeolus* | 2/0/0 | 0.0 | - | - |
| *Otus hoyi* | 0/0/2 | - | - | 0.0 |
| *Otus lempiji* | 0/0/2 | - | - | 0.0 |
| *Otus megalotis* | 0/0/2 | - | - | 0.1 |
| *Otus scops* | 0/0/2 | - | - | 0.0 |
| *Otus sunia* | 0/0/2 | - | - | 0.0 |
| *Oxyura jamaicensis* | 3/0/0 | 0.4 | - | - |
| *Pachyramphus aglaiae* | 2/0/0 | 0.1 | - | - |
| *Pandion haliaetus* | 5/0/0 | 0.0 | - | - |
| *Paradisaea raggiana* | 0/0/2 | - | - | 0.2 |
| *Pardalotus striatus* | 0/0/2 | - | - | 4.3 |
| *Parotia lawesii* | 0/0/2 | - | - | 0.6 |
| *Parula americana* | 7/0/3 | 0.0 | - | 0.0 |
| *Parula gutturalis* | 0/0/4 | - | - | 0.4 |
| *Parula pitiayumi* | 0/0/3 | - | - | 3.8 |
| *Parula superciliosa* | 0/0/3 | - | - | 0.2 |
| *Parus fasciiventer* | 0/0/2 | - | - | 0.8 |
| *Parus major* | 5/0/2 | 1.3 | - | 3.1 |
| *Parus varius* | 0/0/2 | - | - | 6.2 |
| *Passer domesticus* | 6/3/2 | 0.1 | 0.0 | 0.0 |
| *Passer montanus* | 2/0/3 | 0.0 | - | 0.5 |
| *Passerculus sandwichensis* | 7/0/0 | 0.2 | - | - |
| *Passerella iliaca* | 9/0/0 | 0.4 | - | - |
| *Passerina amoena* | 6/0/2 | 0.3 | - | 0.3 |
| *Passerina caerulea* | 0/0/2 | - | - | 0.2 |
| *Passerina ciris* | 3/0/2 | 0.5 | - | 0.1 |
| *Passerina cyanea* | 3/0/3 | 0.2 | - | 0.4 |
| *Passerina leclancherii* | 0/0/2 | - | - | 0.2 |
| *Passerina rositae* | 0/0/2 | - | - | 0.0 |
| *Passerina versicolor* | 4/0/2 | 0.5 | - | 0.1 |
| *Patagioenas fasciata* | 2/0/0 | 0.3 | - | - |
| *Pauxi pauxi* | 0/0/2 | - | - | 0.2 |
| *Pelecanus erythrorhynchos* | 4/0/0 | 0.3 | - | - |
| *Pelecanus occidentalis* | 2/0/0 | 0.0 | - | - |
| *Penelopides manillae* | 0/0/2 | - | - | 2.6 |
| *Penelopides panini* | 0/0/2 | - | - | 0.6 |
| *Perdix perdix* | 6/0/0 | 0.0 | - | - |
| *Periparus ater* | 0/0/2 | - | - | 3.4 |
| *Perisoreus canadensis* | 2/0/0 | 0.3 | - | - |
| *Pernis apivorus* | 0/0/2 | - | - | 14.1 |
| *Petrochelidon fulva* | 2/0/11 | 0.1 | - | 0.7 |
| *Petrochelidon pyrrhonota* | 2/0/0 | 1.5 | - | - |
| *Petrochelidon rufocollaris* | 0/0/2 | - | - | 0.2 |
| *Peucedramus taeniatus* | 3/0/0 | 0.4 | - | - |
| *Phaenicophilus palmarum* | 0/0/2 | - | - | 0.2 |
| *Phaeothlypis fluvicauda* | 0/0/6 | - | - | 4.1 |
| *Phaeothlypis rivularis* | 0/0/3 | - | - | 3.8 |
| *Phaethon rubricauda* | 0/0/3 | - | - | 0.0 |
| *Phainopepla nitens* | 2/0/0 | 0.1 | - | - |
| *Phalacrocorax auritus* | 4/0/0 | 0.0 | - | - |
| *Phalacrocorax brasilianus* | 0/2/0 | - | 0.0 | - |
| *Phalacrocorax carbo* | 4/0/0 | 0.0 | - | - |
| *Phalacrocorax pelagicus* | 9/0/0 | 0.1 | - | - |
| *Phalacrocorax penicillatus* | 5/0/0 | 0.0 | - | - |
| *Phalaenoptilus nuttallii* | 6/0/0 | 0.0 | - | - |
| *Phalaropus lobatus* | 7/0/0 | 0.2 | - | - |
| *Phalaropus tricolor* | 2/0/0 | 0.4 | - | - |
| *Phapitreron leucotis* | 0/0/2 | - | - | 0.0 |
| *Phaps chalcoptera* | 0/0/2 | - | - | 0.4 |
| *Phasianus colchicus* | 2/0/3 | 0.1 | - | 0.7 |
| *Phasianus versicolor* | 0/0/5 | - | - | 1.7 |
| *Pheucticus ludovicianus* | 0/0/2 | - | - | 0.6 |
| *Pheucticus melanocephalus* | 7/0/0 | 0.7 | - | - |
| *Philemon corniculatus* | 0/0/2 | - | - | 0.2 |
| *Philomachus pugnax* | 2/0/0 | 0.1 | - | - |
| *Phodilus badius* | 0/0/2 | - | - | 0.2 |
| *Phoebastria immutabilis* | 2/0/0 | 0.0 | - | - |
| *Phoebastria nigripes* | 2/0/0 | 0.3 | - | - |
| *Phylloscopus affinis* | 0/0/2 | - | - | 0.3 |
| *Phylloscopus borealis* | 2/0/4 | 0.4 | - | 6.4 |
| *Phylloscopus borealis* | 0/0/2 | - | - | 0.4 |
| *Phylloscopus brehmi* | 0/0/2 | - | - | 1.5 |
| *Phylloscopus collybita* | 0/4/12 | - | 0.5 | 1.2 |
| *Phylloscopus fuscatus* | 0/0/7 | - | - | 0.4 |
| *Phylloscopus inornatus* | 0/0/2 | - | - | 1.3 |
| *Phylloscopus maculipennis* | 0/0/2 | - | - | 0.3 |
| *Phylloscopus magnirostris* | 0/0/2 | - | - | 0.7 |
| *Phylloscopus proregulus* | 0/0/2 | - | - | 0.3 |
| *Phylloscopus pulcher* | 0/0/2 | - | - | 1.1 |
| *Phylloscopus schwarzi* | 0/0/2 | - | - | 0.7 |
| *Phylloscopus sibilatrix* | 0/2/2 | - | 0.4 | 1.6 |
| *Phylloscopus trochiloides* | 0/0/2 | - | - | 4.6 |
| *Phylloscopus trochilus* | 0/0/20 | - | - | 0.5 |
| *Pica nuttalli* | 3/0/0 | 0.0 | - | - |
| *Picoides albolarvatus* | 10/0/3 | 0.1 | - | 0.8 |
| *Picoides arcticus* | 4/0/0 | 0.3 | - | - |
| *Picoides arcticus* | 0/0/2 | - | - | 0.1 |
| *Picoides borealis* | 0/0/3 | - | - | 0.4 |
| *Picoides dorsalis* | 4/0/0 | 0.1 | - | - |
| *Picoides mixtus* | 0/0/2 | - | - | 0.1 |
| *Picoides nuttallii* | 6/0/2 | 0.5 | - | 0.0 |
| *Picoides pubescens* | 7/0/2 | 0.1 | - | 0.5 |
| *Picoides scalaris* | 4/0/2 | 0.1 | - | 0.2 |
| *Picoides stricklandi* | 0/0/3 | - | - | 6.3 |
| *Picoides tridactylus* | 2/0/3 | 0.1 | - | 0.5 |
| *Picoides villosus* | 14/0/5 | 0.7 | - | 1.1 |
| *Piculus chrysochloros* | 2/0/2 | 3.4 | - | 5.0 |
| *Piculus flavigula* | 2/0/2 | 2.8 | - | 1.9 |
| *Piculus leucolaemus* | 2/0/2 | 0.3 | - | 0.2 |
| *Piculus rubiginosus* | 3/0/2 | 0.1 | - | 0.1 |
| *Picumnus aurifrons* | 2/0/3 | 0.3 | - | 6.2 |
| *Pinicola enucleator* | 2/0/0 | 0.1 | - | - |
| *Pionus menstruus* | 0/0/3 | - | - | 3.7 |
| *Pipilo chlorurus* | 2/0/0 | 0.0 | - | - |
| *Pipilo crissalis* | 2/0/0 | 0.1 | - | - |
| *Pipilo erythrophthalmus* | 3/0/0 | 0.0 | - | - |
| *Pipilo fuscus* | 2/0/0 | 1.1 | - | - |
| *Pipilo maculatus* | 7/0/0 | 0.1 | - | - |
| *Pipraeidea melanonota* | 0/0/2 | - | - | 0.0 |
| *Piranga bidentata* | 0/0/2 | - | - | 0.1 |
| *Piranga erythrocephala* | 0/0/2 | - | - | 0.1 |
| *Piranga flava* | 0/0/7 | - | - | 4.3 |
| *Piranga leucoptera* | 0/0/3 | - | - | 1.1 |
| *Piranga ludoviciana* | 0/0/4 | - | - | 1.4 |
| *Piranga olivacea* | 3/0/3 | 0.3 | - | 0.5 |
| *Piranga roseogularis* | 0/0/2 | - | - | 0.1 |
| *Piranga rubra* | 3/0/52 | 0.3 | - | 0.5 |
| *Pitangus sulphuratus* | 3/0/0 | 0.1 | - | - |
| *Pithecophaga jefferyi* | 0/0/2 | - | - | 0.0 |
| *Platalea ajaja* | 2/0/0 | 0.3 | - | - |
| *Platycichla leucops* | 0/0/2 | - | - | 0.4 |
| *Platyspiza crassirostris* | 0/2/0 | - | 0.0 | - |
| *Plectrophenax nivalis* | 2/0/41 | 0.1 | - | 0.1 |
| *Plegadis chihi* | 3/0/0 | 0.2 | - | - |
| *Plegadis falcinellus* | 0/0/2 | - | - | 1.6 |
| *Pluvialis dominica* | 4/0/0 | 0.0 | - | - |
| *Pluvialis fulva* | 2/0/0 | 0.0 | - | - |
| *Pluvialis squatarola* | 2/0/0 | 0.1 | - | - |
| *Podiceps auritus* | 2/2/0 | 0.0 | 0.0 | - |
| *Podiceps grisegena* | 3/0/0 | 0.1 | - | - |
| *Podiceps nigricollis* | 2/0/0 | 0.0 | - | - |
| *Podilymbus podiceps* | 5/0/0 | 0.6 | - | - |
| *Poecile montanus* | 0/0/3 | - | - | 6.3 |
| *Poecile palustris* | 0/0/2 | - | - | 0.6 |
| *Poecile atricapillus* | 3/0/0 | 0.3 | - | - |
| *Poecile carolinensis* | 2/0/0 | 0.0 | - | - |
| *Poecile cincta* | 4/0/0 | 0.1 | - | - |
| *Poecile gambeli* | 7/0/2 | 2.0 | - | 5.3 |
| *Poecile niger* | 0/0/2 | - | - | 0.1 |
| *Poecile rufescens* | 7/0/2 | 0.0 | - | - |
| *Poecile rufescens* | 0/0/2 | - | - | 0.0 |
| *Poecile sclateri* | 3/0/0 | 1.2 | - | - |
| *Polemaetus bellicosus* | 0/0/2 | - | - | 0.0 |
| *Polioptila caerulea* | 6/0/2 | 0.2 | - | 2.7 |
| *Polioptila melanura* | 2/0/0 | 0.1 | - | - |
| *Polyborus plancus* | 0/0/2 | - | - | 0.5 |
| *Polyplectron bicalcaratum* | 0/0/2 | - | - | 0.1 |
| *Polysticta stelleri* | 2/0/0 | 0.4 | - | - |
| *Pooecetes gramineus* | 2/0/0 | 0.1 | - | - |
| *Poospiza alticola* | 0/2/0 | - | 0.0 | - |
| *Poospiza melanoleuca* | 0/3/0 | - | 0.4 | - |
| *Poospiza torquata* | 0/2/0 | - | 0.9 | - |
| *Poospiza whitii* | 0/2/0 | - | 2.2 | - |
| *Porphyrio martinica* | 2/0/0 | 0.0 | - | - |
| *Porzana carolina* | 3/0/0 | 0.4 | - | - |
| *Primolius auricollis* | 0/0/2 | - | - | 0.1 |
| *Primolius maracana* | 0/0/2 | - | - | 0.1 |
| *Progne chalybea* | 0/0/2 | - | - | 6.5 |
| *Progne modesta* | 0/0/2 | - | - | 5.7 |
| *Protonotaria citrea* | 5/0/2 | 0.2 | - | 0.2 |
| *Psaltriparus minimus* | 4/0/0 | 2.1 | - | - |
| *Pseudoseisura lophotes* | 0/0/2 | - | - | 0.0 |
| *Psittacula krameri* | 2/0/0 | 0.0 | - | - |
| *Pterodroma brevirostris* | 0/2/0 | - | 0.0 | - |
| *Pterodroma hasitata* | 2/0/0 | 0.1 | - | - |
| *Pteroglossus inscriptus* | 0/0/2 | - | - | 0.0 |
| *Ptilinopus occipitalis* | 0/0/2 | - | - | 0.3 |
| *Puffinus baroli* | 0/0/2 | - | - | 0.0 |
| *Puffinus bulleri* | 6/0/0 | 0.1 | - | - |
| *Puffinus carneipes* | 5/0/0 | 0.0 | - | - |
| *Puffinus creatopus* | 3/0/0 | 0.0 | - | - |
| *Puffinus gravis* | 0/2/0 | - | 0.0 | - |
| *Puffinus mauretanicus* | 0/0/5 | - | - | 0.3 |
| *Puffinus pacificus* | 7/0/0 | 0.1 | - | - |
| *Puffinus puffinus* | 0/0/4 | - | - | 0.8 |
| *Puffinus tenuirostris* | 6/0/0 | 0.0 | - | - |
| *Puffinus yelkouan* | 0/0/9 | - | - | 0.2 |
| *Pycnonotus brunneus* | 0/2/0 | - | 1.5 | - |
| *Pycnonotus plumosus* | 0/4/0 | - | 0.7 | - |
| *Pygoscelis adeliae* | 0/3/0 | - | 0.0 | - |
| *Pygoscelis antarcticus* | 0/0/2 | - | - | 0.0 |
| *Pygoscelis antarcticus* | 0/0/2 | - | - | 8.4 |
| *Pygoscelis papua* | 0/0/2 | - | - | 5.5 |
| *Pyrocephalus rubinus* | 5/0/0 | 0.9 | - | - |
| *Pyrrhura picta* | 0/0/2 | - | - | 0.6 |
| *Quiscalus major* | 5/0/0 | 0.3 | - | - |
| *Quiscalus mexicanus* | 11/0/0 | 0.2 | - | - |
| *Quiscalus quiscula* | 4/0/0 | 0.5 | - | - |
| *Rallus limicola* | 3/0/0 | 0.6 | - | - |
| *Ramphastos brevis* | 0/0/2 | - | - | 0.1 |
| *Ramphastos sulfuratus* | 0/0/2 | - | - | 0.4 |
| *Ramphastos swainsonii* | 0/0/2 | - | - | 0.1 |
| *Ramphastos toco* | 0/0/2 | - | - | 0.5 |
| *Ramphastos tucanus* | 0/0/4 | - | - | 0.5 |
| *Ramphastos vitellinus* | 0/0/8 | - | - | 1.3 |
| *Ramphocelus costaricensis* | 0/0/2 | - | - | 0.1 |
| *Recurvirostra americana* | 2/0/0 | 0.0 | - | - |
| *Regulus calendula* | 5/0/0 | 0.0 | - | - |
| *Regulus regulus* | 0/0/3 | - | - | 6.3 |
| *Regulus satrapa* | 7/0/0 | 0.2 | - | - |
| *Rissa brevirostris* | 2/0/0 | 0.0 | - | - |
| *Rissa tridactyla* | 8/0/0 | 0.1 | - | - |
| *Rostrhamus sociabilis* | 2/0/0 | 0.1 | - | - |
| *Rynchops niger* | 2/0/0 | 0.0 | - | - |
| *Salpinctes obsoletus* | 3/0/0 | 0.2 | - | - |
| *Sarcogyps calvus* | 0/0/2 | - | - | 0.2 |
| *Sayornis nigricans* | 3/0/2 | 0.1 | - | 0.2 |
| *Sayornis phoebe* | 0/0/2 | - | - | 0.1 |
| *Sayornis saya* | 0/0/2 | - | - | 0.1 |
| *Scardafella squammata* | 0/0/2 | - | - | 2.7 |
| *Schistochlamys melanopsis* | 0/0/2 | - | - | 0.4 |
| *Scolopax minor* | 5/0/0 | 0.0 | - | - |
| *Seicercus affinis* | 0/0/5 | - | - | 1.4 |
| *Seicercus burkii* | 0/0/2 | - | - | 0.1 |
| *Seicercus poliogenys* | 0/0/3 | - | - | 4.4 |
| *Seicercus soror* | 0/0/3 | - | - | 0.1 |
| *Seicercus tephrocephalus* | 0/0/2 | - | - | 0.2 |
| *Seicercus valentini* | 0/0/5 | - | - | 0.5 |
| *Seicercus whistleri* | 0/0/5 | - | - | 1.1 |
| *Seiurus aurocapillus* | 8/0/3 | 0.3 | - | 0.3 |
| *Seiurus motacilla* | 3/0/0 | 0.0 | - | - |
| *Selasphorus platycercus* | 3/0/0 | 0.2 | - | - |
| *Selasphorus rufus* | 4/0/0 | 0.1 | - | - |
| *Selasphorus sasin* | 2/0/0 | 0.1 | - | - |
| *Selenidera reinwardtii* | 0/0/2 | - | - | 0.0 |
| *Sericornis frontalis* | 0/2/2 | - | 0.2 | 5.3 |
| *Setophaga ruticilla* | 6/0/2 | 0.2 | - | 0.4 |
| *Sheppardia aequatorialis* | 0/0/2 | - | - | 0.1 |
| *Sheppardia aurantithorax* | 0/0/5 | - | - | 1.0 |
| *Sheppardia cyornithopsis* | 0/0/4 | - | - | 4.8 |
| *Sheppardia gabela* | 0/0/2 | - | - | 2.8 |
| *Sheppardia lowei* | 0/0/3 | - | - | 0.8 |
| *Sheppardia montana* | 0/0/3 | - | - | 0.2 |
| *Sheppardia sharpei* | 0/0/3 | - | - | 0.4 |
| *Sialia currucoides* | 5/0/0 | 0.0 | - | - |
| *Sialia mexicana* | 7/0/0 | 0.4 | - | - |
| *Sialia sialis* | 5/0/3 | 0.2 | - | 0.6 |
| *Sitta carolinensis* | 9/0/0 | 0.4 | - | - |
| *Sitta europaea* | 0/2/0 | - | 1.4 | - |
| *Sitta neumayer* | 0/2/0 | - | 1.3 | - |
| *Sitta pusilla* | 2/0/0 | 0.9 | - | - |
| *Sitta pygmaea* | 5/0/2 | 0.4 | - | 0.0 |
| *Sitta tephronota* | 4/4/0 | 0.2 | 0.0 | - |
| *Sittasomus griseicapillus* | 0/0/2 | - | - | 5.8 |
| *Smicrornis brevirostris* | 0/2/0 | - | 0.0 | - |
| *Smithornis sharpei* | 0/2/0 | - | 0.0 | - |
| *Somateria fischeri* | 7/0/0 | 0.0 | - | - |
| *Somateria mollissima* | 9/0/0 | 0.1 | - | - |
| *Somateria spectabilis* | 4/0/0 | 0.0 | - | - |
| *Sphyrapicus nuchalis* | 5/0/0 | 0.4 | - | - |
| *Sphyrapicus ruber* | 7/0/0 | 0.3 | - | - |
| *Sphyrapicus thyroideus* | 5/0/0 | 0.0 | - | - |
| *Sphyrapicus varius* | 8/0/6 | 1.1 | - | 6.1 |
| *Spindalis zena* | 0/0/3 | - | - | 4.5 |
| *Spizaetus alboniger* | 0/0/3 | - | - | 0.1 |
| *Spizaetus nipalensis* | 0/2/4 | - | 0.0 | 0.9 |
| *Spizaetus ornatus* | 0/0/2 | - | - | 1.1 |
| *Spizaetus tyrannus* | 0/0/3 | - | - | 0.1 |
| *Spizella arborea* | 7/0/0 | 0.0 | - | - |
| *Spizella breweri* | 9/0/7 | 0.1 | - | 0.2 |
| *Spizella pallida* | 5/0/0 | 0.1 | - | - |
| *Spizella passerina* | 7/0/0 | 0.1 | - | - |
| *Spizella pusilla* | 4/0/0 | 0.1 | - | - |
| *Sporophila torqueola* | 2/0/0 | 0.0 | - | - |
| *Stactolaema leucotis* | 0/0/2 | - | - | 0.0 |
| *Stelgidopteryx serripennis* | 2/0/0 | 0.0 | - | - |
| *Stellula calliope* | 4/0/0 | 0.6 | - | - |
| *Stephanoaetus coronatus* | 0/0/2 | - | - | 0.2 |
| *Stercorarius longicaudus* | 5/0/5 | 0.1 | - | 0.1 |
| *Stercorarius parasiticus* | 3/0/5 | 0.3 | - | 5.5 |
| *Stercorarius pomarinus* | 5/0/4 | 0.0 | - | 0.1 |
| *Sterna aleutica* | 5/0/0 | 0.0 | - | - |
| *Sterna anaethetus* | 2/0/0 | 0.0 | - | - |
| *Sterna antillarum* | 2/0/0 | 0.1 | - | - |
| *Sterna caspia* | 3/0/0 | 0.1 | - | - |
| *Sterna elegans* | 5/0/0 | 0.1 | - | - |
| *Sterna forsteri* | 2/0/0 | 0.0 | - | - |
| *Sterna fuscata* | 3/0/0 | 0.1 | - | - |
| *Sterna hirundo* | 2/0/0 | 0.0 | - | - |
| *Sterna maxima* | 4/0/0 | 0.0 | - | - |
| *Sterna nilotica* | 2/0/0 | 0.1 | - | - |
| *Sterna sandvicensis* | 8/0/2 | 0.3 | - | 0.5 |
| *Stiphrornis erythrothorax* | 0/0/2 | - | - | 0.3 |
| *Stiphrornis gabonensis* | 0/0/3 | - | - | 1.1 |
| *Stiphrornis sanghensis* | 0/0/5 | - | - | 0.3 |
| *Stiphrornis xanthogaster* | 0/0/3 | - | - | 0.2 |
| *Streptopelia chinensis* | 0/0/2 | - | - | 0.0 |
| *Streptopelia decaocto* | 2/0/0 | 0.0 | - | - |
| *Streptopelia mayeri* | 0/0/2 | - | - | 0.0 |
| *Strix aluco* | 0/0/14 | - | - | 1.3 |
| *Strix nebulosa* | 4/0/2 | 0.0 | - | 0.0 |
| *Strix occidentalis* | 7/0/0 | 0.0 | - | - |
| *Strix rufipes* | 0/0/2 | - | - | 0.0 |
| *Strix uralensis* | 0/0/2 | - | - | 1.1 |
| *Strix varia* | 4/0/0 | 0.4 | - | - |
| *Strix woodfordii* | 0/0/3 | - | - | 0.1 |
| *Struthio camelus* | 0/4/0 | - | 0.6 | - |
| *Sturnella magna* | 7/0/0 | 2.3 | - | - |
| *Sturnella neglecta* | 4/0/0 | 0.0 | - | - |
| *Sturnus vulgaris* | 10/3/2 | 0.4 | 0.1 | 0.2 |
| *Sula nebouxii* | 0/2/0 | - | 0.0 | - |
| *Surnia ulula* | 0/0/3 | - | - | 0.1 |
| *Swynnertonia swynnertoni* | 0/0/2 | - | - | 1.1 |
| *Sylvia atricapilla* | 0/0/3 | - | - | 0.0 |
| *Sylvia melanocephala* | 3/4/2 | 0.7 | 0.9 | 0.2 |
| *Synthliboramphus antiquus* | 0/0/3 | - | - | 0.1 |
| *Syrmaticus ellioti* | 0/0/5 | - | - | 0.3 |
| *Syrmaticus humiae* | 0/0/3 | - | - | 0.5 |
| *Syrmaticus mikado* | 0/0/2 | - | - | 0.5 |
| *Syrmaticus reevesi* | 0/0/2 | - | - | 0.5 |
| *Tachycineta bicolor* | 7/0/0 | 0.1 | - | - |
| *Taeniopygia guttata* | 0/6/0 | - | 0.1 | - |
| *Tangara arthus* | 0/0/2 | - | - | 3.3 |
| *Tangara chilensis* | 0/0/3 | - | - | 4.3 |
| *Tangara cyanicollis* | 0/0/2 | - | - | 2.0 |
| *Tangara cyanocephala* | 0/0/2 | - | - | 0.0 |
| *Tangara fastuosa* | 0/0/2 | - | - | 0.3 |
| *Tangara gyrola* | 0/0/2 | - | - | 2.4 |
| *Tangara labradorides* | 0/0/2 | - | - | 7.1 |
| *Tangara mexicana* | 0/0/2 | - | - | 3.2 |
| *Tangara nigroviridis* | 0/0/2 | - | - | 1.2 |
| *Tangara punctata* | 0/0/2 | - | - | 6.8 |
| *Tangara ruficervix* | 0/0/2 | - | - | 0.4 |
| *Tangara velia* | 0/0/2 | - | - | 0.1 |
| *Tarsiger cyanurus* | 0/0/2 | - | - | 0.4 |
| *Tauraco persa* | 0/0/2 | - | - | 2.6 |
| *Teretistris fernandinae* | 0/0/2 | - | - | 0.0 |
| *Tetrax tetrax* | 0/0/2 | - | - | 0.0 |
| *Thamnophilus caerulescens* | 0/0/127 | - | - | 0.8 |
| *Thamnophilus unicolor* | 0/0/2 | - | - | 1.4 |
| *Thraupis bonariensis* | 0/0/2 | - | - | 0.0 |
| *Thryomanes bewickii* | 6/0/0 | 2.6 | - | - |
| *Thryothorus ludovicianus* | 4/0/0 | 0.1 | - | - |
| *Tiaris olivacea* | 0/0/2 | - | - | 0.1 |
| *Tockus leucomelas* | 0/0/2 | - | - | 0.4 |
| *Todus angustirostris* | 0/0/3 | - | - | 4.1 |
| *Todus multicolor* | 0/0/3 | - | - | 2.9 |
| *Todus subulatus* | 0/0/3 | - | - | 0.0 |
| *Todus todus* | 0/0/5 | - | - | 10.8 |
| *Torgos tracheliotus* | 0/0/2 | - | - | 8.0 |
| *Toxostoma curvirostre* | 3/0/0 | 4.8 | - | - |
| *Toxostoma lecontei* | 0/0/2 | - | - | 0.1 |
| *Toxostoma redivivum* | 0/0/65 | - | - | 0.4 |
| *Toxostoma rufum* | 6/0/0 | 0.0 | - | - |
| *Trachyphonus darnaudii* | 0/0/2 | - | - | 0.0 |
| *Trachyphonus purpuratus* | 0/0/3 | - | - | 7.1 |
| *Tragopan satyra* | 0/0/2 | - | - | 0.0 |
| *Tragopan temminckii* | 0/0/2 | - | - | 0.4 |
| *Treron vernans* | 0/0/2 | - | - | 0.3 |
| *Tricholaema hirsuta* | 0/0/2 | - | - | 0.3 |
| *Tringa flavipes* | 2/0/0 | 0.0 | - | - |
| *Tringa glareola* | 4/0/0 | 0.1 | - | - |
| *Tringa melanoleuca* | 3/0/0 | 0.0 | - | - |
| *Tringa solitaria* | 10/0/0 | 2.7 | - | - |
| *Troglodytes aedon* | 3/0/0 | 0.2 | - | - |
| *Troglodytes troglodytes* | 7/0/0 | 3.1 | - | - |
| *Turdus chiguanco* | 0/0/2 | - | - | 6.9 |
| *Turdus merula* | 0/0/2 | - | - | 0.3 |
| *Turdus migratorius* | 7/0/2 | 0.1 | - | 0.0 |
| *Turdus naumanni* | 0/2/0 | - | 0.0 | - |
| *Turdus philomelos* | 0/0/2 | - | - | 0.1 |
| *Turdus ruficollis* | 0/2/0 | - | 0.0 | - |
| *Tympanuchus pallidicinctus* | 5/0/0 | 0.0 | - | - |
| *Tympanuchus phasianellus* | 3/0/0 | 0.2 | - | - |
| *Tyrannus crassirostris* | 2/0/0 | 0.0 | - | - |
| *Tyrannus melancholicus* | 2/0/0 | 0.8 | - | - |
| *Tyto alba* | 11/9/0 | 1.8 | 1.6 | - |
| *Uria aalge* | 4/0/2 | 0.9 | - | 1.2 |
| *Uria lomvia* | 3/0/0 | 0.8 | - | - |
| *Veniliornis callonotus* | 3/0/4 | 0.0 | - | 0.1 |
| *Veniliornis dignus* | 2/0/2 | 0.4 | - | 0.8 |
| *Veniliornis frontalis* | 2/0/2 | 0.0 | - | 0.0 |
| *Veniliornis fumigatus* | 2/0/2 | 0.0 | - | 0.4 |
| *Veniliornis nigriceps* | 3/0/2 | 0.0 | - | 0.0 |
| *Veniliornis passerinus* | 2/0/2 | 0.0 | - | 0.1 |
| *Veniliornis spilogaster* | 2/0/2 | 0.0 | - | 0.5 |
| *Vermivora celata* | 4/0/2 | 0.1 | - | 0.7 |
| *Vermivora chrysoptera* | 3/0/2 | 0.0 | - | 0.2 |
| *Vermivora luciae* | 3/0/2 | 0.0 | - | 0.0 |
| *Vermivora peregrina* | 4/0/2 | 0.3 | - | 0.0 |
| *Vermivora pinus* | 3/0/2 | 0.1 | - | 1.7 |
| *Vermivora ruficapilla* | 5/0/4 | 0.9 | - | 1.4 |
| *Vermivora virginiae* | 4/0/3 | 0.0 | - | 0.4 |
| *Vidua chalybeata* | 0/2/0 | - | 0.0 | - |
| *Vireo altiloquus* | 2/0/0 | 0.0 | - | - |
| *Vireo bellii* | 2/0/0 | 0.3 | - | - |
| *Vireo cassinii* | 20/0/0 | 0.4 | - | - |
| *Vireo flavoviridis* | 10/0/2 | 0.1 | - | 0.2 |
| *Vireo gilvus* | 15/0/0 | 1.4 | - | - |
| *Vireo griseus* | 5/0/0 | 0.1 | - | - |
| *Vireo huttoni* | 4/0/0 | 0.3 | - | - |
| *Vireo olivaceus* | 9/0/0 | 0.3 | - | - |
| *Vireo philadelphicus* | 5/0/0 | 0.1 | - | - |
| *Vireo plumbeus* | 2/0/15 | 0.1 | - | 0.5 |
| *Vireo solitarius* | 6/0/8 | 0.6 | - | 0.3 |
| *Vireolanius leucotis* | 0/0/2 | - | - | 5.9 |
| *Vultur gryphus* | 0/2/2 | - | 0.0 | 0.0 |
| *Wilsonia canadensis* | 3/0/2 | 0.2 | - | 0.8 |
| *Wilsonia citrina* | 3/0/0 | 0.1 | - | - |
| *Wilsonia pusilla* | 7/0/0 | 0.6 | - | - |
| *Xanthocephalus xanthocephalus* | 3/0/0 | 0.5 | - | - |
| *Xema sabini* | 2/0/0 | 0.0 | - | - |
| *Xenoligea montana* | 0/0/2 | - | - | 0.5 |
| *Xiphocolaptes promeropirhynchus* | 0/0/2 | - | - | 0.9 |
| *Xiphorhynchus elegans* | 0/0/53 | - | - | 1.2 |
| *Xiphorhynchus erythropygius* | 0/0/3 | - | - | 1.4 |
| *Xiphorhynchus flavigaster* | 0/0/2 | - | - | 1.9 |
| *Xiphorhynchus guttatus* | 0/0/8 | - | - | 5.0 |
| *Xiphorhynchus obsoletus* | 0/0/2 | - | - | 0.3 |
| *Xiphorhynchus ocellatus* | 0/0/4 | - | - | 4.1 |
| *Xiphorhynchus picus* | 0/0/4 | - | - | 1.6 |
| *Xiphorhynchus spixii* | 0/0/24 | - | - | 1.4 |
| *Xiphorhynchus triangularis* | 0/0/3 | - | - | 3.2 |
| *Zeledonia coronata* | 0/0/2 | - | - | 1.0 |
| *Zenaida asiatica* | 2/0/0 | 0.0 | - | - |
| *Zenaida asiatica* | 0/0/2 | - | - | 0.1 |
| *Zenaida auriculata* | 0/0/2 | - | - | 0.0 |
| *Zenaida aurita* | 0/0/2 | - | - | 0.0 |
| *Zenaida galapagoensis* | 0/0/2 | - | - | 0.0 |
| *Zenaida macroura* | 7/0/2 | 0.0 | - | 0.0 |
| *Zonotrichia albicollis* | 6/0/0 | 0.3 | - | - |
| *Zonotrichia atricapilla* | 3/0/0 | 0.0 | - | - |
| *Zonotrichia leucophrys* | 7/0/0 | 0.2 | - | - |
| *Zoothera naevia* | 0/0/2 | - | - | 0.4 |
| *Zoothera princei* | 0/0/2 | - | - | 0.0 |
| *Zosterops japonicus* | 0/2/4 | - | 1.4 | 0.1 |
|  |  |  |  |  |
