# Supplementary material for: Molecular Identification of Birds: Performance of Distance-Based DNA Barcoding in Three Genes to Delimit Parapatric Species
Source: PLoS One. 2009 Jan 7;4(1):e4119. doi: 10.1371/journal.pone.0004119 (PMC2612741; doi:10.1371/journal.pone.0004119)
Supplement: Table S2 — List of all samples that have been sequenced in this study, with voucher numbers and collection localities (0.24 MB DOC) [file pone.0004119.s004.doc]

**Table S2.**

| **Specimen** | **Sample no** | **GenBank accession** | **GenBank**  **accession** | **Locality** |
| --- | --- | --- | --- | --- |
|  |  | ***16S*** | ***COX1*** |  |
| *Acridotheres tristis* | MFUM 200554 | FJ465179 | FJ465290 | Iran |
| *Acridotheres tristis e* | MFUM 200429 | FJ465180 | FJ465291 | Iran |
| *Acrocephalus scirpaceaus fuscus e* | ZMA 56205 | FJ465181 | FJ465292 | Iran |
| *Acrocephalus stentoreus brunnescens* | ZMA 56204 | FJ465182 | FJ465293 | Iran |
| *Alauda arvensis* | MFUM 200467 | FJ465183 | FJ465294 | Iran |
| *Alauda arvensis* | MFUM 200471 | FJ465184 | FJ465295 | Iran |
| *Alauda arvensis* | MFUM 200473 | FJ465184 | FJ465296 | Iran |
| *Alauda arvensis* | MFUM 200474 | FJ465185 | FJ465297 | Iran |
| *Alectoris chukar* | NRM 20036338 |  | FJ465298 | Captive |
| *Alectoris philbyi* | NRM 20036337 | FJ465186 | FJ465299 | Captive |
| *Ammomanes deserti* | MFUM 20045 | FJ465187 | FJ465300 | Iran |
| *Anthus petrosus littoralis* | ZMUC 116892 | FJ465188 | FJ465301 | Denmark |
| *Anthus petrosus littoralis* | ZMUC116893 | FJ465189 | FJ465302 | Denmark |
| *Anthus pratensis pratensis* | NRM 20026012 | FJ465190 | FJ465303 | Sweden |
| *Anthus pratensis pratensis* | ZMUC 116884 | FJ465191 |  | Denmark |
| *Anthus pratensis pratensis* | ZMUC 131790 | FJ465192 |  | Denmark |
| *Anthus spinoletta spinoletta* | ZMUC 116889 | FJ465193 | FJ465304 | Switzerland |
| *Anthus spinoletta spinoletta* | ZMUC 116890 | FJ465194 | FJ465305 | Switzerland |
| *Anthus spinoletta spinoletta* | ZMUC 137278 | FJ465195 | FJ465306 | Italy |
| *Bucanetes githagineus crassirostris* | MFUM 200445 | FJ465196 | FJ465307 | Iran |
| *Bucanetes githagineus crassirostris* | MFUM 200446 | FJ465197 | FJ465308 | Iran |
| *Bucanetes githagineus crassirostris* | ZMA 56207 | FJ465198 | FJ465309 | Iran |
| *Carduelis cannabina bella* | MFUM 200416 | FJ465199 | FJ465310 | Iran |
| *Carduelis carduelis caniceps* | MFUM 200415 | FJ465200 | FJ465311 | Iran |
| *Carduelis carduelis caniceps* | MFUM 200449 | FJ465201 | FJ465312 | Iran |
| *Carduelis carduelis caniceps* | MFUM 200460 | FJ465202 | FJ465313 | Iran |
| *Carduelis flammea* | NRM 20036803 | FJ465203 | FJ465314 | Sweden |
| *Carpospiza brachydactyla* | MFUM 200441 | FJ465204 | FJ465315 | Iran |
| *Carpospiza brachydactyla* | MFUM 200443 | FJ465205 | FJ465316 | Iran |
| *Corvus corax corax* | NRM 2004 6571 | FJ465206 | FJ465317 | Sweden |
| *Corvus monedula monedula* | NRM 20046500 | FJ465207 | FJ465318 | Sweden |
| *Cyanistes caeruleus caeruleus* | NRM 20006358 | FJ465208 |  | Sweden |
| *Dendrocopos syriacus* | NRM20036318 |  | FJ465319 | Captive |
| *Emberiza bruniceps* | ZMA 56199 | FJ465209 | FJ465320 | Iran |
| *Emberiza bruniceps* | ZMA 56200 | FJ465210 | FJ465321 | Iran |
| *Emberiza bruniceps* | ZMA 56198 | FJ465211 | FJ465322 | Iran |
| *Emberiza buchanani cerrutii* | ZMA 56202 | FJ465212 | FJ465323 | Iran |
| *Emberiza caesia* | ZMUC 130563 | FJ465213 |  | Greece |
| *Emberiza caesia* | ZMUC 137275 | FJ465214 |  | Turkey |
| *Emberiza cia* | MFUM 200418 | FJ465215 | FJ465324 | Iran |
| *Emberiza cia* | MFUM 200556 | FJ465216 | FJ465325 | Iran |
| *Emberiza hortulana* | ZMA 56201 | FJ465217 | FJ465326 | Iran |
| *Emberiza hortulana* | ZMUC 116260 | FJ465218 |  | Denmark |
| *Emberiza hortulana* | ZMUC 116261 | FJ465219 |  | Denmark |
| *Emberiza melanocephala* | ZMA56197 | FJ465220 | FJ465327 | Iran |
| *Eremophila alpestris albigula* | MFUM 20042 | FJ465221 | FJ465328 | Iran |
| *Eremophila bilopha* | ZMUC 135715 | FJ465222 | FJ465329 | Tunisia |
| *Ficedula albicollis* | NRM 20036504 | FJ465223 | FJ465330 | Sweden |
| *Ficedula parva* | ZMA 56210 | FJ465224 | FJ465331 | Iran |
| *Galerida cristata* | MFUM 200442 | FJ465225 | FJ465332 | Iran |
| *Galerida theklae superflua* | ZMUC 131967 | FJ465226 |  | Tunis |
| *Hippolais icterina icterina* | ZMUC 117807 | FJ465227 | FJ465333 | Denmark |
| *Hippolais icterina icterina* | ZMUC 117808 | FJ465228 | FJ465334 | Denmark |
| *Hippolais icterina icterina* | ZMUC 119833 | FJ465229 |  | Tanzania |
| *Hippolais icterina icterina* | ZMUC 137197 | FJ465230 | FJ465335 | Denmark |
| *Hippolais icterina icterina* | ZMUC 117806 |  | FJ465336 | Poland |
| *Hippolais polyglotta* | ZMUC 133863 | FJ465231 |  | Denmark |
| *Lanius isabellinus* | ZMA 56203 | FJ465232 | FJ465337 | Iran |
| *Lanius collurio* | NRM 20046428 | FJ465233 | FJ465338 | Sweden |
| *Lanius meridionalis aucheri* | MFUM 200411 | FJ465234 |  | Iran |
| *Lanius minor* | MFUM 200422 | FJ465235 | FJ465339 | Iran |
| *Larus argentatus argentatus* | ZMUC 112578 | FJ465236 |  | Denmark |
| *Larus argentatus argentatus* | ZMUC 130665 | FJ465237 | FJ465340 | Denmark |
| *Larus cachinnans cachinnans* | ZMUC 136951 | FJ465238 | FJ465341 | Russia |
| *Locustella fluviatilis* | ZMUC 131388 | FJ465239 |  | Kenya |
| *Locustella lanceolata* | ZMUC 117812 | FJ465240 |  | China |
| *Locustella naevia naevia* | ZMUC 117814 | FJ465241 |  | Denmark |
| *Luscinia luscinia* | NRM 20026317 | DQ683442* | DQ683476* | Sweden |
| *Luscinia megarhynchos hafizi* | MFUM 200359 | DQ683443* | DQ683477* | Iran |
| *Melanocorypha bimaculata torquata* | ZMA 56192 | FJ465242 | FJ465342 | Iran |
| *Melanocorypha calandra psammochroa* | ZMA 56191 | FJ465243 | FJ465343 | Iran |
| *Monticola solitarius* | MFUM 20044 | FJ465244 | FJ465344 | Iran |
| *Motacilla alba* | NRM 20016303 | FJ465245 | FJ465345 | Sweden |
| *Oenanthe alboniger* | MIUT2003-7.2(28) | DQ683444* | DQ683478* | Iran |
| *Oenanthe alboniger* | MIUT2003-104(29) | DQ683445* | DQ683479* | Iran |
| *Oenanthe alboniger* | MIUT2003-95(18) | DQ683446* | DQ683480* | Iran |
| *Oenanthe chrysopygia* | MIUT2003-96(19) | DQ683447* | DQ683481* | Iran |
| *Oenanthe d. deserti* | MIUT2003-3(33) | DQ683448* | DQ683482* | Iran |
| *Oenanthe d. deserti* | BMNH A/2005.2.5 | DQ683449* | DQ683483* | Iran |
| *Oenanthe d. deserti* | MIUT2003-98(21) | DQ683450* | DQ683484* | Iran |
| *Oenanthe deserti homochroa* | MIUT2003-99(22) | DQ683451* | DQ683485* | Morocco |
| *Oenanthe finschii barnesi* | MIUT2003-91(14) | DQ683452* | DQ683486* | Iran |
| *Oenanthe finschii barnesi* | BMNH A/2005.2.11 | DQ683453* | DQ683487* | Iran |
| *Oenanthe finschii barnesi* | MIUT2003-100(23) | DQ683454* | DQ683488* | Iran |
| *Oenanthe isabellina* | MIUT2003-84(7) | DQ683456* | DQ683490* | Iran |
| *Oenanthe isabellina* | BMNH A/2005.2.12 | DQ683457* | DQ683491* | Iran |
| *Oenanthe isabellina* | BMNH A/2005.2.1 | DQ683458* | DQ683492* | Iran |
| *Oenanthe isabellina* | BMNH A/2005.2.2 | DQ683459* | DQ683493* | Iran |
| *Oenanthe isabellina* | BMNH A/2005.2.3 | DQ683460* | DQ683494* | Iran |
| *Oenanthe isabellina* | MIUT2003-90(13) | DQ683461* | DQ683495* | Iran |
| *Oenanthe leucopyga aegra* | MIUT2003-137 | DQ683474* | DQ683508* | Morocco |
| *Oenanthe lugens persica* | BMNH A/2005.2.6 | DQ683462* | DQ683496* | Iran |
| *Oenanthe lugens persica* | BMNH A/2005.2.7 | DQ683463* | DQ683497* | Iran |
| *Oenanthe lugens persica* | BMNH A/2005.2.8 | DQ683464* | DQ683498* | Iran |
| *Oenanthe lugens persica* | MIUT2003-94(17) | DQ683465* | DQ683499* | Iran |
| *Oenanthe moesta moesta* | MIUT2003-103(26) | DQ683466* | DQ683500* | Iran |
| *Oenanthe oenanthe libanotica* | BMNH A/2005.2.9 | DQ683467* | DQ683501* | Iran |
| *Oenanthe oenanthe libanotica* | BMNH A/2005.2.4 | DQ683468* | DQ683502* | Iran |
| *Oenanthe oenanthe libanotica* | MIUT2003-81 | DQ683469* | DQ683503* | Iran |
| *Oenanthe oenanthe libanotica* | BMNH A/2005.2.10 | DQ683470* | DQ683504* | Iran |
| *Oenanthe oenanthe seebohmi* | MIUT2003-83 | DQ683471* | DQ683505* | Morocco |
| *Oenanthe pleschanka* | MIUT2003-102(25) | DQ683472* | DQ683506* | Iran |
| *Oenanthe pleschanka* | MIUT2003-26(30) | DQ683473* | DQ683507* | Iran |
| *Oenathe pleschanka×Oenanthe hispanica* | MIUT2003-37(32) | DQ683455* | DQ683489* | Iran |
| *Oenanthe picata picata* | MIUT2003-7.1(27) | DQ683475* | DQ683509* | Iran |
| *Oriolus oriolus* | MFUM 20041 | FJ465246 | FJ465346 | Iran |
| *Parus major* | ZMUC 133916 |  | FJ465347 | Greece |
| *Parus major major* | ZMUC 134022 |  | FJ465348 | Sweden |
| *Parus major major* | ZMUC 134036 |  | FJ465349 | Sweden |
| *Parus major* | ZMUC 135484 |  | FJ465350 | Greece |
| *Parus major* | ZMUC 136952 | FJ465247 | FJ465351 | Russia |
| *Passer domesticus* | MFUM 200432(39 | FJ465248 | FJ465352 | Iran |
| *Passer domesticus* | MFUM 200436(41 | FJ465249 | FJ465353 | Iran |
| *Passer indicus* | ZMUC 117474 | FJ465250 |  | Pakistan |
| *Phylloscopus affinis* | ZMUC 117832 | FJ465251 |  | China |
| *Phylloscopus bonelli* | ZMUC 121484 | FJ465252 | FJ465354 | Senegal |
| *Phylloscopus collybita* | NRM 20046497 | FJ465253 | FJ465355 | Sweden |
| *Phylloscopus collybita* | ZMUC 137138 | FJ465254 |  | Denmark |
| *Phylloscopus collybita abietinus* | ZMUC 131383 | FJ465255 |  | Kenya |
| *Phylloscopus collybita tristis* | ZMUC 117866 | FJ465256 |  | China |
| *Phylloscopus sibilatix* | ZMUC 129907 | FJ465257 |  | Denmark |
| *Phylloscopus sibilatix* | ZMUC 137263 | FJ465258 | FJ465356 | Italy |
| *Phylloscopus trochilus acredula* | ZMA 56206 | FJ465259 | FJ465357 | Iran |
| *Ptyonoprogne rupestrisi* | ZMA 56211 | FJ465260 | FJ465358 | Iran |
| *Rhodospiza obsolete* | MFUM 200450 |  | FJ465359 | Iran |
| *Sitta neumayer* | MFUM245 | FJ465261 | FJ465360 | Iran |
| *Sitta neumayer neumayer* | ZMUC 129871 | FJ465262 |  | Greece |
| *Sitta tephronota iranica* | ZMA56193 | FJ465263 | FJ465361 | Iran |
| *Sitta tephronota iranica* | ZMA56194 | FJ465264 | FJ465362 | Iran |
| *Sitta tephronota iranica* | ZMA56195 | FJ465265 | FJ465363 | Iran |
| *Sitta tephronota iranica* | MFUM 200440 | FJ465266 | FJ465364 | Iran |
| *Sturnus vulgaris* | MFUM 200431 | FJ465267 | FJ465365 | Iran |
| *Sturnus unicolor* | ZMUC 119334 | FJ465268 |  | Spain |
| *Sylvia communis* | ZMA 562087 | FJ465269 | FJ465366 | Iran |
| *Sylvia curruca curruca* | NRM 20026345 | FJ465270 | FJ465367 | Sweden |
| *Sylvia m. melanocephala* | ZMUC 131876 | FJ465271 | FJ465368 | Cyprus |
| *Sylvia m. melanocephala* | ZMUC 131969 | FJ465272 |  | Greece |
| *Sylvia m. melanocephala* | ZMUC 131992 | FJ465273 | FJ465369 | Cyprus |
| *Sylvia m. melanocephala* | ZMUC 132073 | FJ465274 | FJ465370 | Spain |
| *Sylvia mystacea* | ZMA 56209 | FJ465275 | FJ465371 | Iran |
| *Turdus atrogularis* | NRM 19980930 | FJ465276 |  | China |
| *Turdus naumanni* | NRM 20000926 | FJ465277 | FJ465372 | China |
| *Turdus naumanni* | NRM 20000927 | FJ465278 |  | China |
| *Turdus pallidus* | NRM 19991106 |  | FJ465373 | China |
| *Turdus ruficollis* | NRM 20050609 | FJ465279 |  | China |
| *Turdus torquatus* | MFUM 200555 | FJ465280 | FJ465374 | Iran |
| *Tyto alba* | ZMA 55945 | FJ465281 | FJ465375 | Netherlands Antilles |
| *Tyto alba* | ZMA 58257 | FJ465282 | FJ465376 | Netherlands Antilles |
| *Tyto alba* | ZMA 58259 | FJ465283 | FJ465377 | Netherlands Antilles |
| *Tyto alba bargei* | ZMA 55939 | FJ465284 | FJ465378 | Netherlands Antilles |
| *Tyto alba bargei* | ZMA 55941 | FJ465285 | FJ465379 | Netherlands Antilles |
| *Tyto alba bargei* | ZMA 55942 | FJ465286 | FJ465380 | Netherlands Antilles |
| *Tyto alba bargei* | ZMA 55943 | FJ465287 | FJ465381 | Netherlands Antilles |
| *Tyto alba guttata* | ZMA 58235 | FJ465288 | FJ465382 | Netherlands |
| *Tyto alba guttata* | ZMA 58237 | FJ465289 | FJ465383 | Netherlands |

Museums acronomys: MFUM, Museum of Ferdowsi University of Mashhad; NRM, Swedish Museum of Natural History; ZMUC, Zoological Museum, University of Copenhagen; ZMA, Zoological Museum of the University of Amsterdam. *our published sequences (Aliabadian *et al.* 2007).
